# ZRANB2/SNHG20/FOXK1 Axis regulates Vasculogenic mimicry formation in glioma

**DOI:** 10.1186/s13046-019-1073-7

**Published:** 2019-02-11

**Authors:** Xiaozhi Li, Yixue Xue, Xiaobai Liu, Jian Zheng, Shuyuan Shen, Chunqing Yang, Jiajia Chen, Zhen Li, Libo Liu, Jun Ma, Teng Ma, Yunhui Liu

**Affiliations:** 1 Department of Neurosurgery, Shengjing Hospital of China Medical University, Shenyang, 110004 People’s Republic of China; 2 Liaoning Clinical Medical Research Center in Nervous System Disease, Shenyang, 110004 China; 3 Key Laboratory of Neuro-oncology in Liaoning Province, Shenyang, 110004 China; 4 Department of Neurobiology, School of Life Sciences, China Medical University, Shenyang, 110122 China; 5 Key Laboratory of Cell Biology, Ministry of Public Health of China, China Medical University, Shenyang, 110122 China; 6 Key Laboratory of Medical Cell Biology, Ministry of Education of China, China Medical University, Shenyang, China

**Keywords:** ZRANB2, SNHG20, FOXK1, Glioma, Vasculogenic mimicry formation

## Abstract

**Background:**

Glioma is the most common intracranial neoplasm with vasculogenic mimicry formation as one form of blood supply. Many RNA-binding proteins and long non-coding RNAs are involved in tumorigenesis of glioma.

**Methods:**

The expression of ZRANB2, SNHG20 and FOXK1 in glioma were detected by real-time PCR or western blot. The function of ZRANB2/SNHG20/FOXK1 axis in glioma associated with vasculogenic mimicry formation was analyzed.

**Results:**

ZRANB2 is up-regulated in glioma tissues and glioma cells. ZRANB2 knockdown inhibits the proliferation, migration, invasion and vasculogenic mimicry formation of glioma cells. ZRANB2 binds to SNHG20 and increases its stability. Knockdown of SNHG20 reduces the degradation of FOXK1 mRNA by SMD pathway. FOXK1 inhibits transcription by binding to the promoters of MMP1, MMP9 and VE-Cadherin and inhibits vasculogenic mimicry formation of glioma cells.

**Conclusions:**

ZRANB2/SNHG20/FOXK1 axis plays an important role in regulating vasculogenic mimicry formation of glioma, which might provide new targets of glioma therapy.

**Electronic supplementary material:**

The online version of this article (10.1186/s13046-019-1073-7) contains supplementary material, which is available to authorized users.

## Introduction

Glioma is globally recognized as the most common primary intracranial neoplasm [1, 2]. Despite the existence of various treatment methods including surgery, radiation and chemotherapy, the median survival time of patients suffering glioma is no more than 15 months [3, 4]. Although glioma tissue is characterized by angiogenesis and vasculogenesis [5], tumor treatment effects of anti-angiogenic drugs including bevacizumab are far from satisfaction [6, 7]. Vasculogenic mimicry (VM) formation was first discovered in 1999 and regarded as a new form of blood supply independent of blood vessels [8]. The study of VM formation may bring light to the treatment of glioma.

RNA-binding protein (RBPs) complexes are one class of proteins binding specifically to certain RNAs to form RNA-binding proteins (RNPs), which can regulate transcription, editing, alternative splicing, polyadenylation, translocation, etc. Considering these variable functions, RBPs are expected as important targets for cancer treatment [9]. ZRANB2 (zinc-finger RAN-binding domain containing protein 2) is one kind of RNA-binding proteins originally identified in rat juxtaglomerular cells [10]. ZRANB2 could inhibit the BMP (bone morphogenetic proteins) signaling pathway by binding to Smad protein in HEK293T cells [11]. ZRANB2 was also reported highly expressed in ovarian serous papillary carcinoma [10]. However, no report of ZRANB2 expression in glioma tissues and cells and involvement in the regulation of VM formation has been reported.

Long non-coding RNAs (LncRNAs) are non-coding RNA molecules with a total length of more than 200 nucleotides. Recent studies have shown that lncRNAs regulate gene expression in epigenetic regulation, transcriptional regulation, post-transcriptional regulation and translational regulation [12], which have potential value in diagnosis and treatment of glioma. SNHG20 was originally identified in hepatocellular carcinoma, localized to 17q25.2, and highly expressed in hepatocellular carcinoma, promoting hepatocellular carcinoma proliferation and migration, and was negatively correlated with patient prognosis [13]. It also played a cancer-promoting role in colorectal cancer, non-small cell lung cancer, cervical cancer, and breast cancer [14–17]. There are no reports of SNHG20 in regulating glioma VM.

The Staufen1 (STAU1)-mediated mRNA decay (SMD) pathway is one of the ways in which lncRNAs degrade mRNAs in mammalian cells. The Alu element of lncRNAs can form the STAU1 binding site (SBS) by specifically binding to the Alu element in the 3’UTR of the target gene. The target gene mRNA is prone to recruit the RNA helicase and ATPase frameshift increase protein 1 (UPF1), forming the complex STAU1-UPF1 which allows the degradation of target gene mRNA [18, 19].

The transcription factor FOXK1 (Forkhead box K1, FOXK1) is an important member of the forkhead family of proteins. Studies have shown that FOXK1 has different levels of expression in different tumors and plays different roles. FOXK1 was highly expressed in colorectal cancer, and FOXK1 and FOXK2 transfered DVL (Dishevelled)-related proteins into the nucleus, which positively regulated Wnt/β-catenin signaling pathway [20]. However, FOXK1 works as a tumor suppressor in breast cancer, and its expression was positively correlated with the prognosis of breast cancer [21]. At present, the regulation of VM in glioma by FOXK1 is unknown.

Matrix metalloproteinase 1 (MMP1) and matrix metalloproteinase 9 (MMP9) belong to the family of metalloproteinases that degrade extracellular matrix. It has been demonstrated that MMP1 and MMP9 were positively associated with VM formation in clear cell renal carcinoma and ovarian cancer, respectively [22, 23]. Vascular endothelial cadherin (VE-Cadherin) had been shown to be highly expressed in gliomas and was associated with tumor malignancy and promotes vasculogenic mimicry formation in glioma [24]. There has been no report whether FOXK1 regulates transcription of MMP1, MMP9 and VE-Cadherin and regulates the VM formation in glioma.

This study first examined the endogenous expression of ZRANB2, SNHG20 and FOXK1 in glioma tissues and U87 and U251 glioma cells, and studied the effects of ZRANB2, SNHG20 and FOXK1 on VM formation in glioma. Then we explored the probable molecular mechanism of ZRANB2, SNHG20 and FOXK1 in regulating VM formation. This study aims to provide new theoretical basis for the study of the mechanism of glioma development and provide new targets for the treatment of glioma.

## Methods

### Human tissue samples

Human glioma tissues and normal brain tissues (NBTs) were acquired from patients diagnosed with glioma undergoing neurosurgeries at Shengjing Hospital of China Medical University. All patients voluntarily signed informed consent before surgery and the consent was permitted by the Ethics Committee of Shengjing Hospital of China Medical University. Surgical resection specimens were placed in liquid nitrogen immediately after isolation before use. The pathological grades were determined by the pathologists according to WHO classification and were divided into low-grade glioma (WHO I-II: *n* = 15) and high-grade glioma (WHO III-IV: *n* = 15). Normal brain tissues acquired from patients undergoing traumatic surgeries without any clear brain diseases before (*n* = 15) were used as negative controls.

### Cell culture

The human glioma cell lines (U87 and U251) and human embryonic kidney cell line (HEK293T) were purchased from Shanghai Institutes for Biological Sciences Cell Resource Center. Dulbecco’s modified Eagle medium (DMEM)/high glucose with 10% FBS was used to culture U87 and HEK293T cells while DMEM/F12 medium with 10% FBS was used to culture U251 cells. Normal human astrocyte (NHA) cells (Catalog #1800, 1 million cells per vial) were purchased from ScienCell Research Laboratories (Carlsbad, CA, USA) and were cultured in RPMI-1640 medium. All the cells were maintained in a humidified incubator at 37 °C with 5% CO_2_.

### Quantitative real-time PCR (qRT-PCR)

Total RNA was extracted from human tissue samples and HA, U87 and U251 cells using Trizol reagent (Life Technologies Corporation, Carlsbad, CA). RNA quality and concentration were measured via 260/280 nm absorbance with Nanodrop Spectrophotometer (ND-100, Thermo, Waltham, MA). SNHG20, FOXK1 mRNA, GAPDH mRNA concentration were detected using One-Step SYBR PrimeScript RT-PCR Kit (TakaraBio, Inc., Japan) in 7500 Fast RT-PCR System (Applied Biosystems, USA). The relative quantification (2^-△△Ct^) was calculated after the expression levels were normalized using GAPDH as endogenous control.

### Western blotting

Total proteins were gained after cells were lysed in RIPA buffer (Beyotime Institute of Biotechnology) for 30 min and were then centrifuged at 17,000 g at 4 °C for 45 min. Protein concentrations were determined by BCA protein assay kit (Beyotime Institute of Biotechnology, China). Protein samples were subjected to SDS-PAGE and electrophoretically transferred to PVDF membranes. After incubated in Tween-Tris-buffered saline (TTBS) containing 5% nonfat milk at room temperature for 2 h, membranes were then incubated with primary antibodies as follows: ZRANB2 (1:500, Proteintech, Rosemont, IL), FOXK1 (1:200, Santa Cruz, USA), MMP1 (1:500, Proteintech, Rosemont, IL), MMP9 (1:500, Proteintech, Rosemont, IL), VE-Cadherin (1:500, Affinity Biosciences, USA), GAPDH (1:10000, Proteintech, Rosemont, IL). After washed three times with TTBS, membranes were incubated with horseradish peroxidase conjugated secondary antibodies at room temperature for 2 h. Immunoblots were visualized with ECL (enhanced chemiluminescence) Kit (Beyotime Institute of Biotechnology, China) and scanned using ChemImager 5500 V2.03 software (Alpha Innotech, San Leandro, CA). Integrated density values (IDVs) were calculated using GAPDH as an internal control.

### Cell transfections

Short-hairpin RNA against ZRANB2 (ZRANB2(−)), short-hairpin RNA against FOXK1 (FOXK1(−)), short-hairpin RNA against STAU1 (STAU1(−)), short-hairpin RNA against UPF1 (UPF1(−)), ZRANB2 full length plasmid (ZRANB2(+)) and their respective non-targeting sequence (negative control, NC) were synthesized (GenePharma, Shanghai, China). Short-hairpin RNA against SNHG20 (SNHG20(−)) and its respective non-targeting sequence (negative control, NC) were synthesized (GeneChem, Shanghai, China). SNHG20 full length plasmid (SNHG20(+)) and FOXK1 full length plasmid (FOXK1(+)) and their respective non-targeting sequence (negative control, NC) were synthesized (GeneScript, Piscataway, Nj, USA). FOXK1 sequence with assumptive SNHG20 binding site was amplified by PCR and cloned into pmirGLO Dual-Luciferase Vector (GenePharama, China) as wild type (FOXK1–3’UTR-Wt). The corresponding FOXK1 sequence without assumptive SNHG20 binding site (GenePharama, China) was cloned into pmirGLO Dual-Luciferase Vector as mutant type (FOXK1–3’UTR-Mut). Cells were seeded in 24-well plates (Corning, NJ, USA) and transfected using Lipofectamine 3000 reagent (Life Technologies Corporation, Carlsbad, CA) according to its instructions. The stable transfected cells were selected using G418 (0.4 mg/ml), puromycin (1 μg/ml) or hygromycin (500 μg/ml). The silencing and over-expressions efficiency were measured by qRT-PCR. To investigate the effect of ZRANB2 on glioma, cells were divided into five groups: control, ZRANB2(−)-NC, ZRANB2(−), ZRANB2(+)-NC and ZRANB2(+) groups. To investigate the effect of SNHG20 on glioma, cells were divided into five groups: control, SNHG20(−)-NC, SNHG20(−), SNHG20(+)-NC and SNHG20(+) groups. To explore whether SNHG20 mediated the regulation of ZRANB2 affecting the behaviors of glioma, cells were divided into five groups: control, ZRANB2(−) + SNHG20(−), ZRANB2(−) + SNHG20(+), ZRANB2(+) + SNHG20(−), ZRANB2(+) + SNHG20(+) groups. To investigate the effect of FOXK1 on glioma, cells were divided into five groups: control, FOXK1(−)-NC, FOXK1(−), FOXK1(+)-NC and FOXK1(+) groups. To explore whether FOXK1 mediated the regulation of SNHG20 affecting the behaviors of glioma, cells were divided into five groups: control, SNHG20(−) + FOXK1(−), SNHG20(−) + FOXK1(+), SNHG20(+) + FOXK1(−) and SNHG20(+) + FOXK1(+) groups.

### Cell proliferation assay

Cell Counting Kit-8 (CCK-8, Beyotime Institute of Biotechnology, China) assay was used to evaluate the proliferation of cells. Cells were seeded in 96-well plates at the density of 2000 cells per well. After 72 h, 10 μl CCK-8 solution was added and cells were incubated at 37 °C for 2 h. The absorbance was measured at 450 nm on the SpectraMax M5 microplate reader (Molecular Devices, USA).

### Cell migration and invasion assay

Cells were resuspended in 100 μl serum-free medium at the density of 2 × 10^5^ cells per milliliter and seeded in the upper chamber for migration (or precoated with 500 ng/ml Matrigel solution (BD, Franklin Lakes, NJ) for invasion) with 8 μm pore size polycarbonate membrane (Corning, NY, USA) while 600 μl medium containing 10% FBS was added to the lower chamber. 24 h later, cells migrated or invaded to the lower side of membrane surface were fixed and stained 20% Giemsa. Three random fields under a microscope were chosen to count cell numbers.

### In vitro VM tube formation assay

24-well plates were coated with 300 μl Matrigel solution per well and placed at 37 °C for 30 min. Cells were resuspended and seeded with serum-free medium at a density of 3 × 10^5^ cells per well in the Matrigel-coated wells and incubated at 37 °C for more than 24 h. Photos of cells were taken under the inverted microscope (Olympus, Tokyo, Japan). Numbers of VM tube structures in three random fields were counted.

### CD34-periodic acid-schiff (PAS) dual-staining

5 μm formalin-fixed and paraffin-embedded tissue specimens underwent dewaxed in xylene, hydrated in gradient ethanol and boiled in EDTA antigen-unmasking solution. After cooled gradually at room temperature, the tissue specimens were incubated with peroxide, blocked with goat serum and incubated with CD34 primary monoclonal antibody (1:50, Proteintech, Rosemont, IL) at 4 °C for 16 h. After incubation with secondary antibody at 37 °C for 10 min, the tissue specimens were stained with DAB kit (MaiXin Biotech, China). Periodic acid solution, schiff solution and hematoxylin were used for the next PAS staining. VM density was counted under microscope in five random fields.

### Chromatin immunoprecipitation assay

Chromatin immunoprecipitation (CHIP) kit (Beyotime Institute of Biotechnology, China) was used according to the manufacturer’s protocol. 1 × 10^7^ cells were cross-linked with 1% formaldehyde for 10 min and then treated with glycine at room temperature for 5 min. Cells were lysed in buffer solution containing PMSF and resuspended. Chromatin was digested by micrococcal nuclease. Immunoprecipitates were incubated with 3 μg anti-FOXK1 antibody (Santa Cruz, USA) or normal rabbit IgG and incubated with Protein G agarose beads at 4 °C overnight with gentle shaking while 2% lysates were used as input reference. DNA crosslink was reversed with 5 M NaCl and proteinase K at 65 °C for 2 h. Then the DNA was purified and amplified by PCR using the following primers: one of putative binding site of FOXK1 in MMP1 promoter using the primers 5′- TGAGTAAGATATCAGTCTTGACGCA - 3′ and 5′- GTGTCTCCCACCTTTCCCAC - 3′, generating a 101 bp product; the other putative binding site of FOXK1 in MMP1 promoter using the primers 5′- CCTAGCACCAAGGAGCGAAG -3′ and 5′- CCGGATGATGAAAAGGCTGG - 3′, generating a 107 bp product; control group of putative binding site FOXK1 in MMP1 promoter using the primers 5′- TGCTTGTCATAAGGGGTAAAGGA -3′ and 5′- ACCAAATCCTAGCAATGCCT -3′, generating a 177 bp product; the putative binding site of FOXK1 in MMP9 promoter using the primers 5′- AGATCACGCCACTGCACTC -3′ and 5′- CGGGCAGGGTCTATATTCACC - 3′, generating a 147 bp product; control group of putative binding site FOXK1 in MMP9 promoter using the primers 5′- GACAGCCCCAAGTGCCAATA -3′ and 5′- CCCCCACTTGCCATCAATG -3′, generating a 101 bp product; the putative binding site of FOXK1 in VE-Cadherin promoter using the primers 5′- GGATGACACAACTGGCCAGA -3′ and 5′- GACTCCAGCTCTAAGGTGCC - 3′, generating a 119 bp product; control group of putative binding site FOXK1 in VE-Cadherin promoter using the primers 5′- AGGAGTCCCAAGGAGAGCTT -3′ and 5′- TGATGGGGTCAGAATGGCTG -3′, generating a 141 bp product.

### Reporter vector constructs and luciferase reporter assay

HEK293T cells were seed in 96-well plates and co-transfected with FOXK1–3’UTR-Wt (or FOXK1–3’UTR-Mut) reporter plasmid and SNHG20(+)-NC or SNHG20(+), respectively. The relative luciferase activities were measured 48 h after co-transfection using Dual-Luciferase reporter assay kit (Promega).

### RNA immunoprecipitation

EZ-Magna RNA-binding protein immunoprecipitation kit (Millipore, USA) was used according to the manufacture’s protocol. 3 × 10^7^ cell lysate was incubated with RIP buffer with magnetic beads conjugated with 5 μg human anti-Ago2 or normal mouse IgG (as negative control). Samples were incubated with Proteinase K and immunoprecipitated RNA was then isolated. The concentration of RNA was measured by spectrophotometer (NanoDrop, USA). The RNA samples were purified and analyzed by qRT-PCR.

### Nascent RNA capture

Click-iT® Nascent RNA Capture Kit (Thermo Fisher Scientific, USA) was used to detect nascent according to the manufacture’s protocol. Briefly, nascent RNAs were marked with 0.2 mM 5-ethymyl uridine (EU) and the EU-nascent RNA was captured on magnetic beads for subsequent qRT-PCR.

### mRNA stability assay

Cells culture medium were added 5 μg/ml actinomycin D (ActD, NobleRyder, China) to inhibit the de novo RNA synthesis. Total RNA was extracted at 0, 3, 6, 9, 12, 15 h and its concentrations were measured by qRT-PCR. The half-life of RNA was determined by its level at certain point of time compared with time zero.

### Xenograft mouse model in vivo

For the vivo study, 4-week-old athymic nude mice (BALB/c) were purchased from the Cancer Institute of the China Academy of Medical Science and randomly divided into five groups: control, ZRANB2(−)-NC + SNHG20(−)-NC, ZRANB2(−), SNHG20(−) and ZRANB2(−) + SNHG20(−) groups. All the experiments with mice were conducted strictly in accordance with the Animal Welfare Act approved by the Ethics Committee of China Medical University.

For subcutaneous xenografts, 3 × 10^5^ glioma cells were injected into the right flank area of nude mice. Tumor volumes were measured every 5 days until ultimate 45 days according to the estimate formula: volume (mm^3^) = length × width^2^/2. VM of nude mice xenograft model was detected by CD34-PAS dual-staining later. For survival analysis, 3 × 10^5^ glioma cells were stereotactically infected to the right striatum of nude mice. Kaplan-Meier survival curve was applied for the survival analysis.

### Statistical analysis

All descriptive data are expressed as mean ± SD. SPSS 19.0 software was used for statistical analysis with Student’s *t*-test or one-way ANOVA. Differences were considered statistically significant if *P* < 0.05.

## Results

### ZRANB2 was up-regulated in glioma tissues and cells, and knockdown of ZRANB2 inhibited VM formation

**Fig. 1:**
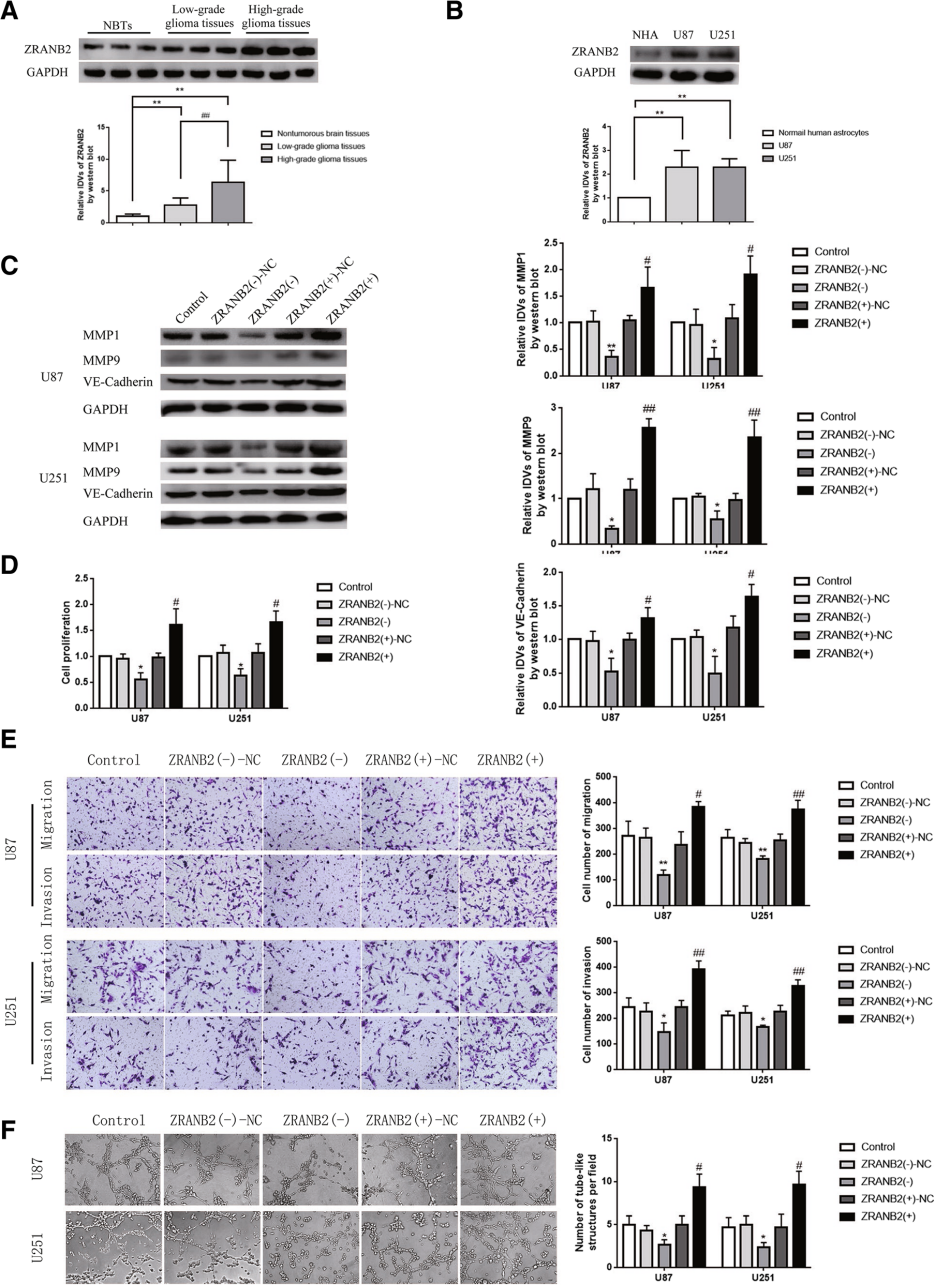
Endogenous expression of ZRANB2 and effect of ZRANB2 on biological behaviors of glioma cells. **a** Protein levels of ZRANB2 in NBTs, low-grade human glioma tissues (LGGTs) and high-grade human glioma tissues (HGGTs). Representative protein expressions and corresponding IDVs of ZRANB2 in NBTs (*n* = 15), low-grade glioma (*n* = 15), and high-grade glioma (*n* = 15) are shown; data are presented as mean ± SD.^**^P < 0.01 vs. NBTs group, ^##^*P* < 0.01 vs. LGGTs group. **b** Protein levels of ZRANB2 in NHA, U87 and U251 cells. Representative protein expressions and corresponding IDVs of ZRANB2 in NHA, U87 and U251 are shown; data are presented as mean ± SD (*n* = 3, each group). ^**^P < 0.01 vs. NHA group. **c** Protein levels of MMP1, MMP9 and VE-Cadherin regulated by ZRANB2 in U87 and U251 cells. Representative protein expressions and corresponding IDVs of MMP1, MMP9, VE-Cadherin in U87 and U251 are shown; data are presented as mean ± SD (*n* = 3, each group). ^*^P < 0.05 vs. ZRANB2(−)-NC group, ^**^P < 0.01 vs. ZRANB2(−)-NC group, ^#^*P* < 0.05 vs. ZRANB2(+)-NC group, ^##^*P* < 0.01 vs. ZRANB2(+)-NC group. **d** CCK-8 assay was used to measure the effect of proliferation on U87 and U251 cells by ZRANB2. Data are presented as mean ± SD (*n* = 3, each group). ^*^P < 0.05 vs. ZRANB2(−)-NC group, ^#^*P* < 0.05 vs. ZRANB2(+)-NC group. **e** Transwell assay was used to measure the effect of migration and invasion on U87 and U251 cells by ZRANB2. Representative images and corresponding statistical plots are shown. Data are presented as mean ± SD (*n* = 3, each group). ^*^P < 0.05 vs. ZRANB2 (−)-NC group, ^**^P < 0.01 vs. ZRANB2(−)-NC group, ^#^*P* < 0.05 vs. ZRANB2(+)-NC group, ^##^*P* < 0.01 vs. ZRANB2(+)-NC group. Scale bars indicate 50 μm. **f** Three-dimensional culture was used to measure the effect of VM on U87 and U251 cells by ZRANB2. Representative images and corresponding statistical plots are shown. Data are presented as mean ± SD (*n* = 3, each group). ^*^P < 0.05 vs. ZRANB2(−)-NC group, ^**^P < 0.01 vs. ZRANB2(−)-NC group, ^#^*P* < 0.05 vs. ZRANB2(+)-NC group. Scale bars indicate 50 μm

The expression of ZRANB2 in glioma tissues as well as glioma cell lines U87 and U251 was detected by western blotting. As shown in Fig. 1a-b, the expression of ZRANB2 in glioma tissues of different grades was up-regulated compared with normal brain tissues, and was positively correlated with pathological grade of glioma. Besides, the level of ZRANB2 in U87 and U251 was higher than NHA. To further explore the functional role of ZRANB2 in gliomas, we assessed the effects of ZRANB2 knockdown and overexpression on proliferation, migration, invasion and vasculogenic mimicry formation of U87 and U251 cells. As shown in Fig. 1c, the expression of VM-related molecular MMP1, MMP9, VE-Cadherin was down-regulated in ZRANB2(−) group compared with ZRANB2(−)-NC group, while up-regulated in ZRANB2(+) group compared with ZRANB2(+)-NC group. Cell proliferation in ZRANB2(−) group was decreased compared with ZRANB2(−)-NC group, while increased in ZRANB2(+) group compared with ZRANB2(+)-NC group (Fig. 1d). In addition, migrating and invading cell numbers in ZRANB2(−) group was reduced compared with ZRANB2(−)-NC group, while increased in ZRANB2(+) group compared with ZRANB2(+)-NC group (Fig. 1e). Moreover, the ability of VM formation of cells was down-regulated in ZRANB2(−) group compared with ZRANB2(−)-NC group, while up-regulated in ZRANB2(+) group compared with ZRANB2(+)-NC group (Fig. 1f). To validate the correlation between VM and pathological grade of glioma, we conducted the CD34-PAS dual-staining. As shown in Additional file 1: Figure S1A, VM was significantly associated with glioma grade.

### SNHG20 was up-regulated in glioma tissues and cells, and knockdown of SNHG20 inhibited VM formation

**Fig. 2:**
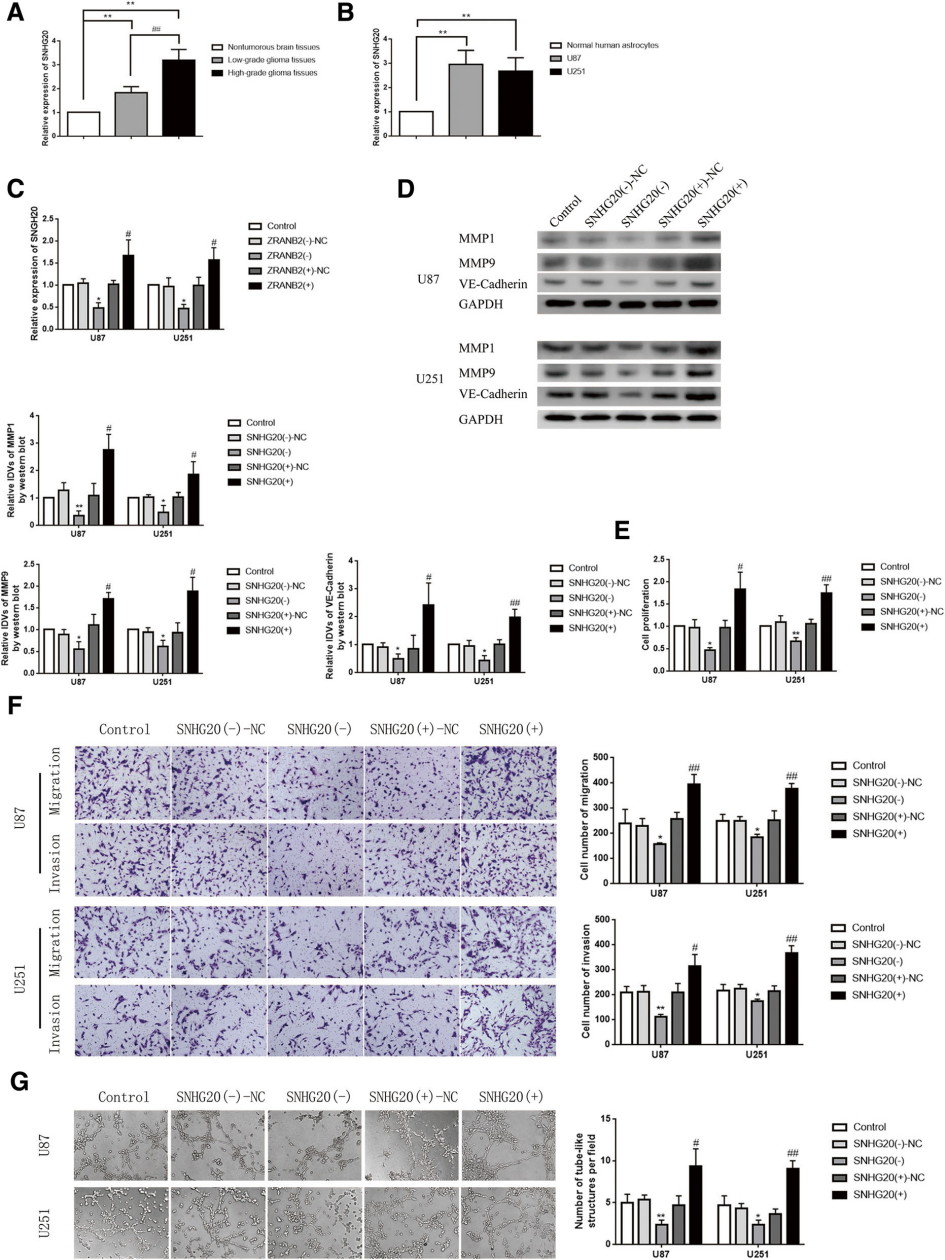
Endogenous expression of SNHG20 and effect of SNHG20 on the biological behaviors of glioma cells. **a** Expression levels of SNHG20 in NBTs, LGGTs and HGGTs. Data are presented as mean ± SD (NBTs (*n* = 15), LGGTs (*n* = 15), HGGTs (*n* = 15)). ^**^P < 0.01 vs. NBTs group, ^##^*P* < 0.01 vs. LGGTs group. **b** Expression of SNHG20 in NHA, U87 and U251 cells. Data are presented as mean ± SD (*n* = 3, each group). ^**^P < 0.01 vs. NHA group. **c** Expression levels of SNHG20 regulated by ZRANB2 in U87 and U251 cells. Data are presented as mean ± SD (*n* = 3, each group). ^*^P < 0.05 vs. ZRANB2(−)-NC group, ^#^*P* < 0.05 vs. ZRANB2(+)-NC group. **d** Protein levels of MMP1, MMP9 and VE-Cadherin regulated by SNHG20 in U87 and U251 cells. Representative protein expressions and corresponding IDVs of MMP1, MMP9, VE-Cadherin in U87 and U251 are shown; data are presented as mean ± SD (*n* = 3, each group). ^*^P < 0.05 vs. SNHG20(−)-NC group, ^**^P < 0.01 vs. SNHG20(−)-NC group, ^#^*P* < 0.05 vs. SNHG20(+)-NC group, ^##^*P* < 0.01 vs. SNHG20(+)-NC group. **e** CCK-8 assay was used to measure the effect of proliferation on U87 and U251 cells by SNHG20. Data are presented as mean ± SD (*n* = 3, each group). ^*^P < 0.05 vs. SNHG20(−)-NC group, ^**^P < 0.01 vs. SNHG20(−)-NC group, ^#^*P* < 0.05 vs. SNHG20(+)-NC group, ^##^*P* < 0.01 vs. SNHG20(+)-NC group. **f** Transwell assay was used to measure the effect of migration and invasion on U87 and U251 cells by SNHG20. Representative images and corresponding statistical plots are shown. Data are presented as mean ± SD (*n* = 3, each group). ^*^P < 0.05 vs. SNGH20(−)-NC group, ^**^P < 0.01 vs. SNHG20(−)-NC group, ^#^*P* < 0.05 vs. SNHG20(+)-NC group, ^##^*P* < 0.01 vs. SNHG20(+)-NC group. Scale bars indicate 50 μm. **g** Three-dimensional culture was used to measure the effect of VM on U87 and U251 cells by SNHG20. Representative images and corresponding statistical plots are shown. Data are presented as mean ± SD (*n* = 3, each group). ^*^P < 0.05 vs. SNHG20(−)-NC group, ^#^*P* < 0.05 vs. SNHG20(+)-NC group. Scale bars indicate 50 μm

The expression of SNHG20 in glioma tissues as well as glioma cell lines U87 and U251 was detected by qRT-PCR. As shown in Fig. 2a-b, the expression of SNHG20 in glioma tissues of different grades was up-regulated compared with NBTs and was positively correlated with pathological grade of glioma; the expression of SNHG20 in U87 and U251 was also increased compared with NHA. In addition, we detected the expression of SNHG20 in cells treated with ZRANB2 knockdown and overexpression. As shown in Fig. 2c, the level of SNHG20 was reduced in ZRANB2(−) group compared with ZRANB2(−)-NC group while increased in ZRANB2(+) group compared with ZRANB2(+)-NC group. We further analyze the functional role of ZRANB2 knockdown and overexpression in gliomas. As shown in Fig. 2d, the expression of MMP1, MMP9, VE-Cadherin was down-regulated in SNHG20(−) group compared with SNHG20(−)-NC group, while up-regulated in SNHG20(+) group compared with SNHG20(+)-NC group. Cell proliferation in SNHG20(−) group was decreased compared with SNHG20(−)-NC group, while increased in SNHG20(+) group compared with SNHG20(+)-NC group (Fig. 2e). In addition, migration and invasion abilities of glioma cells in SNHG20(−) group was inhibited compared with SNHG20(−)-NC group, while enhanced in SNHG20 (+) group compared with SNHG20(+)-NC group (Fig. 2f). As shown in Fig. 2g, the ability of VM formation was down-regulated in SNHG20(−) group compared with SNHG20(−)-NC group, while up-regulated in SNHG20(+) group compared with SNHG20(+)-NC group.

### ZRANB2 promoted VM formation in glioma cells by increasing the stability of SNHG20

**Fig. 3:**
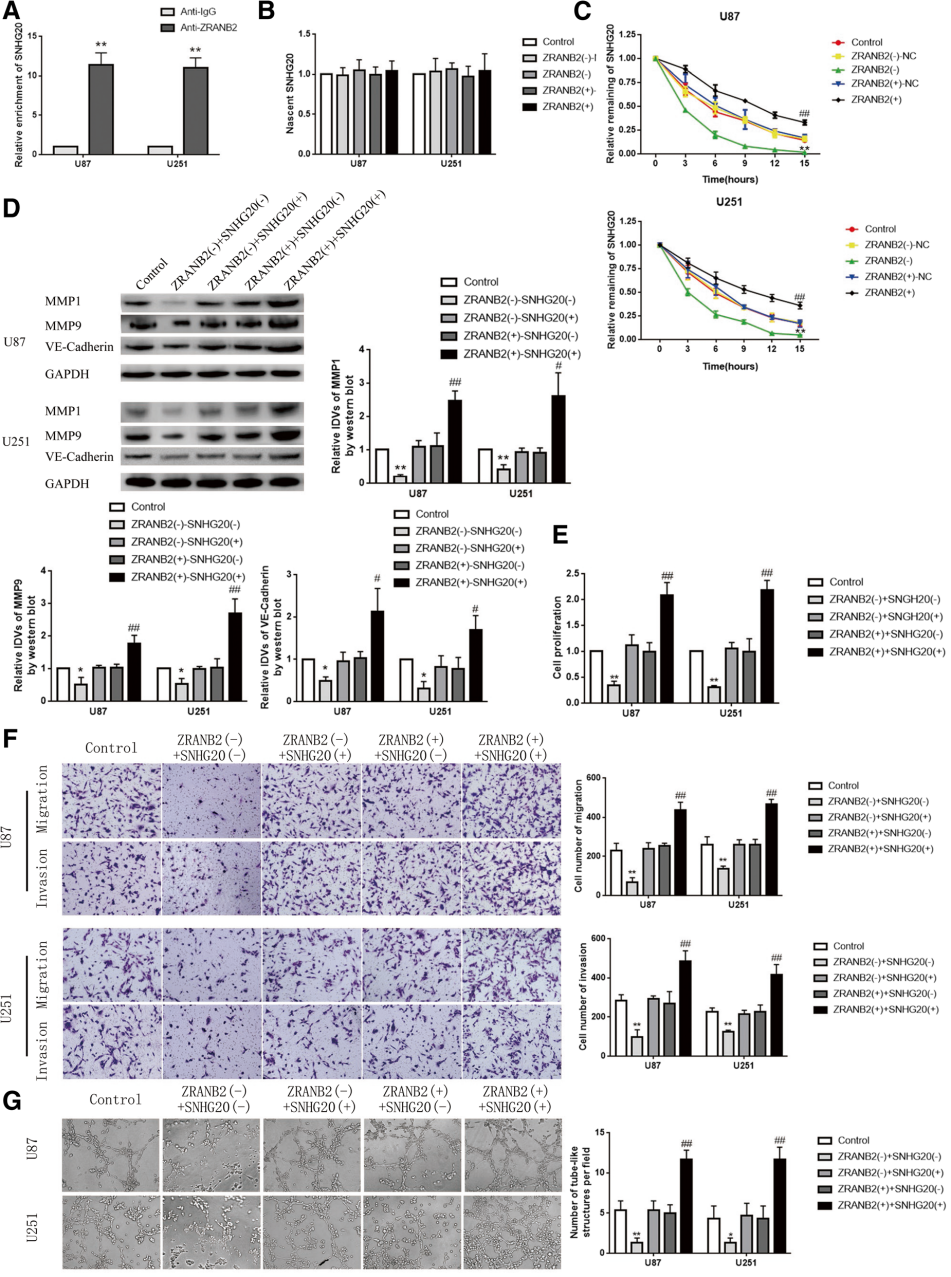
ZRANB2 strengthened the malignant behaviors of glioma cells by stabilizing SNHG20. **a** RNA-IP confirmed the binding interaction between ZRANB2 and SNGH20. Relative enrichment was measured by qRT-PCR; data are presented as mean ± SD (*n* = 3, each group). ^**^P < 0.01 vs. anti-normal IgG respective group. **b** The graph shows nascent SNHG20 in U87 and U251 cells; data are presented as mean ± SD (n = 3, each group). **c** The graph shows SNHG20 levels at different times treated by ActD in U87 and U251 cells (regulated by ZRANB2); data are presented as mean ± SD (*n* = 3, each group). ^**^P < 0.01 vs. ZRANB2(−)-NC group, ^##^*P* < 0.01 vs. ZRANB2(+)-NC group. **d** Protein levels of MMP1, MMP9 and VE-Cadherin regulated by ZRANB2 and SNHG20 in U87 and U251 cells. Representative protein expressions and corresponding IDVs of MMP1, MMP9, VE-Cadherin in U87 and U251 are shown; data are presented as mean ± SD (*n* = 3, each group). ^*^P < 0.05 vs. ZRANB2(−) + SNHG20(+) group, ^**^P < 0.01 vs. ZRANB2(−) + SNHG20(+) group, ^#^*P* < 0.05 vs. ZRANB2(+) + SNHG20(−) group, ^##^*P* < 0.01 vs ZRANB2(+) + SNHG20(−) group. **e** CCK-8 assay was used to measure the effect of proliferation on U87 and U251 cells by ZRANB2 and SNHG20. Data are presented as mean ± SD (*n* = 3, each group). ^**^P < 0.01 vs. ZRANB2(−) + SNHG20(+) group, ^##^*P* < 0.01 vs. ZRANB2(+) + SNHG20(−) group. **f** Transwell assay was used to measure the effect of migration and invasion on U87 and U251 cells by ZRANB2 and SNHG20. Representative images and corresponding statistical plots are shown. Data are presented as mean ± SD (*n* = 3, each group). ^**^P < 0.01 vs. ZRANB2(−) + SNHG20(+) group, ^##^*P* < 0.01 vs ZRANB2(+) + SNHG20(−) group. Scale bars indicate 50 μm. **g** Three-dimensional culture was used to measure the effect of VM on U87 and U251 cells by ZRANB2 and SNHG20. Representative images and corresponding statistical plots are shown. Data are presented as mean ± SD (*n* = 3, each group). ^*^P < 0.05 vs. ZRANB2(−) + SNHG20(+) group, ^#^*P* < 0.05 vs. ZRANB2(+) + SNHG20(−) group. Scale bars indicate 50 μm

To explore the correlation between ZRANB2 and SNHG20, we further used RNA-IP assay to detect whether ZRANB2 directly bound with SNHG20. As shown in Fig. 3a, the enrichment of SNHG20 in the anti-ZRANB2 group was higher than the anti-IgG group. We further analyzed the nascent SNHG20 and half-life of SNHG20 in glioma cells treated with ZRANB2 knockdown and overexpression. There was no significant statistical difference of nascent SNHG20 between ZRANB2(−) and ZRANB2(−)-NC as well as ZRANB2(+) and ZRANB2(+)-NC (Fig. 3b). However, half-life of SNHG20 was reduced in ZRANB2(−) group compared with ZRANB2(−)-NC group while was increased in ZRANB2(+) group compared with ZRANB2(+)-NC group (Fig. 3c). We further constructed co-transfection of ZRANB2(−) and ZRANB2(+) cells with SNHG20(−) and SNHG20(+). As shown in Fig. 3d-g, co-transfection of ZRANB2(−) cells with SNHG20(−) could strongly decrease the expression of MMP1, MMP9, VE-Cadherin and strongly inhibit the abilities of proliferation, migration, invasion and VM of glioma cells. In addition, transfection with SNHG20(+) could rescued the inhibitory effect of ZRANB2(−) on proliferation, migration, invasion and VM of glioma cells, while SNHG20(−) could rescued the promoting effect of ZRANB2(+) similarly.

### FOXK1 was down-regulated in glioma tissues and cells, and overexpression of FOXK1 inhibited VM formation

**Fig. 4:**
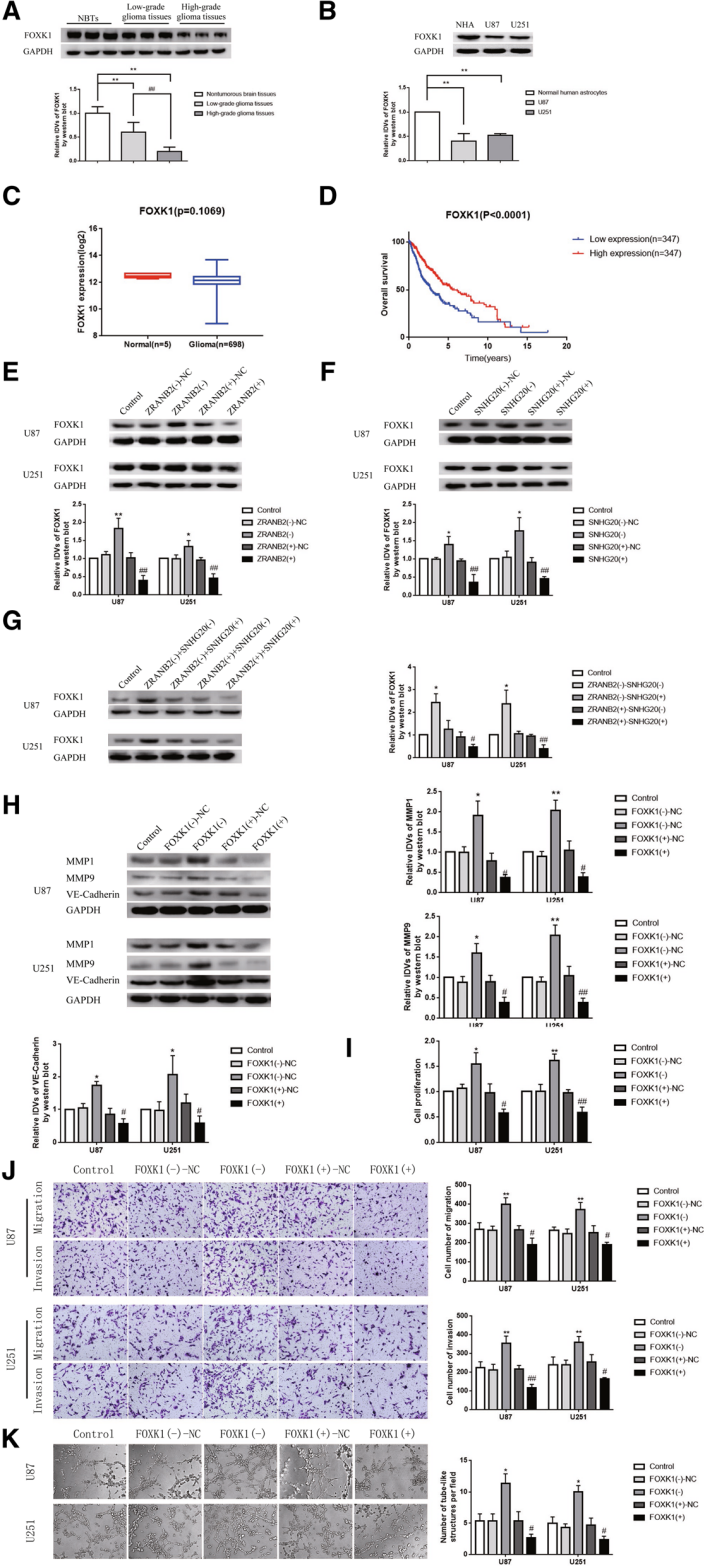
Endogenous expression of FOXK1 and effect of FOXK1 on the biological behaviors of glioma cells. **a** Protein levels of FOXK1 in NBTs, LGGTs and HGGTs. Representative protein expressions and corresponding IDVs of FOXK1 in NBTs (*n* = 15), low-grade glioma (*n* = 15), and high-grade glioma (*n* = 15) are shown; data are presented as mean ± SD.^**^P < 0.01 vs. NBTs group, ^##^*P* < 0.01 vs. LGGTs group. **b** Protein levels of FOXK1 in NHA, U87 and U251 cells. Data are presented as mean ± SD (*n* = 3, each group). ^**^P < 0.01 vs. NHA group. **c** Gene expression difference curve according to TCGA. **d** Survival analysis curve according to TCGA. **e** Effects of ZRANB2 on FOXK1 expression; data are presented as mean ± SD (*n* = 3, each group). ^*^P < 0.05 vs. ZRANB2(−)-NC group, ^**^P < 0.01 vs. ZRANB2(−)-NC group, ^##^*P* < 0.01 vs. ZRANB2(+)-NC group. **f** Effects of SNHG20 on FOXK1 expression; data are presented as mean ± SD (*n* = 3, each group). ^*^P < 0.05 vs. SNHG20(−)-NC group, ^##^*P* < 0.01 vs. SNHG20(+)-NC group. **g** FOXK1 expression regulated by ZRANB2 and SNHG20; data are presented as mean ± SD (*n* = 3, each group). ^*^P < 0.05 vs. ZRANB2(−) + SNHG20(+) group, ^#^*P* < 0.05 vs. ZRANB2(+) + SNHG20(−) group, ^##^*P* < 0.01 vs. ZRANB2(+) + SNHG20(−) group. **h** Protein levels of MMP1, MMP9 and VE-Cadherin regulated by FOXK1. Data are presented as mean ± SD (n = 3, each group). ^*^P < 0.05 vs. FOXK1(−)-NC group, ^**^P < 0.01 vs. FOXK1(−)-NC group, ^#^*P* < 0.05 vs. FOXK1(+)-NC group, ^##^*P* < 0.01 vs. FOXK1(+)-NC group. **i** CCK-8 assay. Data are presented as mean ± SD (*n* = 3, each group). ^*^P < 0.05 vs. FOXK1(−)-NC group, ^**^P < 0.01 vs. FOXK1(−)-NC group, ^#^*P* < 0.05 vs. FOXK1(+)-NC group, ^##^*P* < 0.01 vs. FOXK1(+)-NC group. **j** Transwell assay. Representative images and corresponding statistical plots are shown. Data are presented as mean ± SD (*n* = 3, each group). ^**^P < 0.01 vs. FOXK1(−)-NC group, ^#^*P* < 0.05 vs. FOXK1(+)-NC group, ^##^*P* < 0.01 vs. FOXK1(+)-NC group. Scale bars indicate 50 μm. **k** Three-dimensional culture. Data are presented as mean ± SD (*n* = 3, each group). ^*^P < 0.05 vs. FOXK1(−)-NC group, ^#^*P* < 0.05 vs. FOXK1 (+)-NC group. Scale bars indicate 50 μm

The expression of FOXK1 in glioma tissues as well as U87 and U251 was detected by western blotting. As shown in Fig. 4a-b, the expression of FOXK1 in glioma tissues was down-regulated compared with NBTs and was negatively correlated with pathological grade of glioma. The level of FOXK1 in U87 and U251 was lower than NHA. We also queried the Cancer Genome Atlas database (TCGA), evaluated the association between FOXK1 and glioma, and drew the corresponding survival curve. As shown in Fig. 4c-d, compared with the NBTs, the expression of FOXK1 was down-regulated in the glioma group, but there were only 5 cases in the NBTs group and the statistical difference was not so significant (*P* = 0.1069). However, glioma with high FOXK1 expression had significant longer survival time than those with low FOXK1 expression (*P* < 0.0001). We also investigated the roles of ZRANB2 and SNHG20 on the expression of FOXK1. As shown in Fig. 4e-g, the expression of FOXK1 in ZRANB2(−) and SNHG20(−) was increased compared with ZRANB2(−)-NC and SNHG20(−)-NC group respectively, while the expression of FOXK1 in ZRANB2(+) and SNHG20(+) was decreased compared with ZRANB2(+)-NC and SNHG20(+)-NC group respectively. Meanwhile, transfection with SNHG20(+) could rescued the promoting effect of the expression of FOXK1 by ZRANB2(−), while SNHG20(−) could rescued the inhibitory effect by ZRANB2(+) similarly. Further, we constructed cells for FOXK1 overexpression and knockdown to analyze the function of FOXK1. As shown in Fig. 4h, the expression of MMP1, MMP9, VE-Cadherin was up-regulated in FOXK1(−) group compared with FOXK1(−)-NC group, while down-regulated in FOXK1(+) group compared with FOXK1(+)-NC group. Moreover, VM formation was up-regulated in FOXK1(−) group compared with FOXK1(−)-NC group, while down-regulated in FOXK1(+) group compared with FOXK1(+)-NC group (Fig. 4i-k).

### SNHG20 promoted degradation of FOXK1 through SMD pathway and enhanced VM formation in glioma cells

**Fig. 5:**
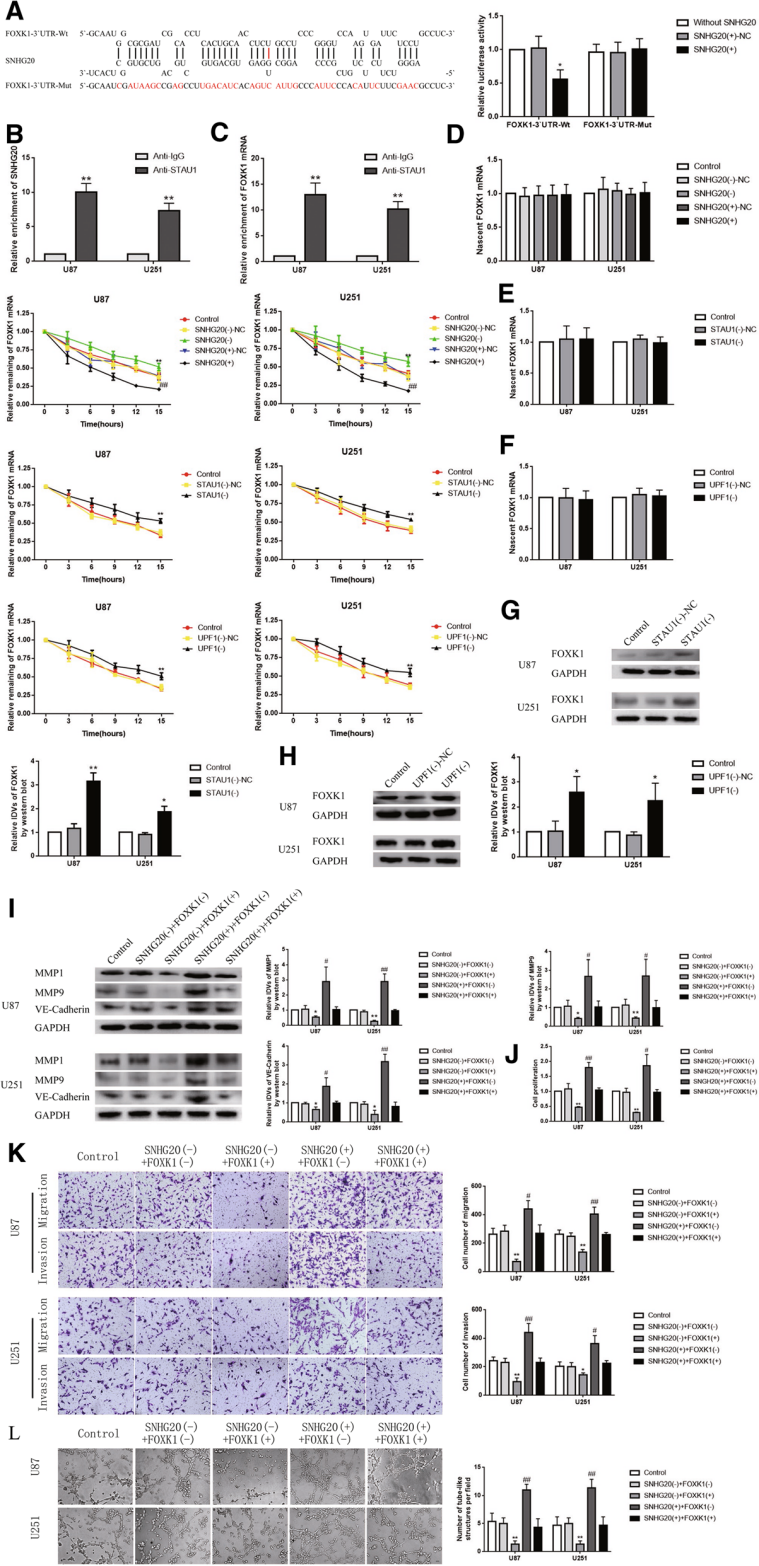
SNHG420 strengthened the malignant behaviors of glioma cells by degrading FOXK1 mRNA through SMD pathway. **a** The predicted SNHG20 binding site in FOXK1 mRNA 3’UTR and results of dual-luciferase reporter assays. Data are presented as mean ± SD (*n* = 3, each group). ^*^P < 0.05 vs. FOXK1–3’UTR-Wt + SNHG20(+)-NC group. **b** RNA-IP results. Data are presented as mean ± SD (*n* = 3, each group). ^**^P < 0.01 vs. anti-normal IgG group. **c** RNA-IP results. Data are presented as mean ± SD (*n* = 3, each group). ^**^P < 0.01 vs. anti-normal IgG group. **d** Stability of FOXK1 mRNA by SNHG20; data are presented as mean ± SD (*n* = 3, each group). ^**^P < 0.01 vs. SNHG20(−)-NC group, ^##^*P* < 0.01 vs. SNHG20(+)-NC group. **e** Stability of FOXK1 mRNA by STAU1; data are presented as mean ± SD (*n* = 3, each group). ^**^P < 0.01 vs. SNHG20(−)-NC group. **f** Stability of FOXK1 mRNA by UPF1; data are presented as mean ± SD (*n* = 3, each group). ^**^P < 0.01 vs. SNHG20(−)-NC group. **g** Effect of STAU1 on FOXK1 expression; data are presented as mean ± SD (*n* = 3, each group). ^*^P < 0.05 vs. STAU1(−)-NC group, ^**^P < 0.01 vs. STAU1(−)-NC group. **h** Effect of UPF1 on FOXK1 expression; data are presented as mean ± SD (*n* = 3, each group). ^*^P < 0.05 vs. UPF1(−)-NC group. **i** Protein levels of MMP1, MMP9 and VE-Cadherin. Data are presented as mean ± SD (*n* = 3, each group). ^*^P < 0.05 vs. SNHG20(−) + FOXK1(−) group, ^**^P < 0.01 vs. SNHG20(−) + FOXK1(−) group, ^#^*P* < 0.05 vs. SNHG20(+) + FOXK1(+) group, ^##^*P* < 0.01 vs. SNHG20(+) + FOXK1(+) group. (J) CCK-8. Data are presented as mean ± SD (*n* = 3, each group). ^**^P < 0.01 vs. FOXK1(−)-NC group, ^#^*P* < 0.05 vs. FOXK1(+)-NC group, ^##^*P* < 0.01 vs. FOXK1(+)-NC group. **k** Transwell. Data are presented as mean ± SD (*n* = 3, each group). ^*^P < 0.05 vs. SNHG20(−) + FOXK1(−) group, ^**^P < 0.01 vs. SNHG20(−) + FOXK1(−) group, ^#^*P* < 0.05 vs. SNHG20(+) + FOXK1(+) group, ^##^*P* < 0.01 vs. SNHG20(+) + FOXK1(+) group. Scale bars indicate 50 μm. **l** Three-dimensional culture. Data are presented as mean ± SD (*n *= 3, each group). ^*^P < 0.05 vs. SNHG20(−) + FOXK1(−) group, ^**^P < 0.01 vs. SNHG20(−) + FOXK1(−) group, ^##^*P* < 0.01 vs. SNHG20(+) + FOXK1(+) group. Scale bars indicate 50 μm

Using the bioinformatics database (IntaRNA), we found FOXK1 mRNA might be a putative target of SNHG20 in a sequence-specific manner. To verify our hypothesis, we used dual-luciferase gene reporter assays to confirm the predicted binding site. As shown in Fig. 5a, the relative luciferase activity in the FOXK1–3’UTR-Wt + SNHG20(+) group was significantly reduced compared with the FOXK1–3’UTR-Wt + SNHG20(+)-NC group, while there was no significant difference between FOXK1–3’UTR-Mut + SNHG20(+) and FOXK1–3’UTR-Mut + SNHG20(+)-NC group. Furthermore, the relative enrichment of SNHG20 (FOXK1 mRNA) and STAU1 in the Ago2 group was higher than the IgG group (Fig. 5b-c). To determine whether STAU1 and UPF1 involved in the interaction between SNHG20 and FOXK1 mRNA, U87 and U251 cells were transfected with STAU1 knockdown plasmid and UPF1 knockdown plasmid. As shown in Fig. 5d-f, there was no significant difference of nascent FOXK1 mRNA between SNHG20(−), SNHG20(+), STAU1(−), UPF1(−) group and SNHG20(−)-NC, SNHG20(+)-NC, STAU1(−)-NC, UPF1(−)-NC group respectively. Moreover, the half-life of FOXK1 mRNA was increased in SNHG20(−), STAU1(−), UPF1(−) group compared with SNHG20(−)-NC, STAU1(−)-NC, UPF1(−)-NC group respectively, while was reduced in SNHG20(+) group compared with SNHG20(+)-NC group. In addition, the expression of FOXK1 was up-regulated in the STAU1(−) and UPF1(−) group compared with STAU1(−)-NC and UPF1(−)-NC group respectively (Fig. 5g-h). As shown in Fig. 5i-l, co-transfection of SNHG20(−) cells with FOXK1(+) strongly decreased the expression of MMP1, MMP9, VE-Cadherin and strongly inhibited the abilities of proliferation, migration, invasion and VM of glioma cells. In addition, transfection with FOXK1(−) could rescue the inhibitory effect of SNHG20(−) on proliferation, migration, invasion and VM of glioma cells, while FOXK1(+) could rescue the promoting effect of SNHG20 (+) similarly.

### FOXK1 bound to the promoters of MMP1, MMP9 and VE-cadherin and inhibited transcription

**Fig. 6:**
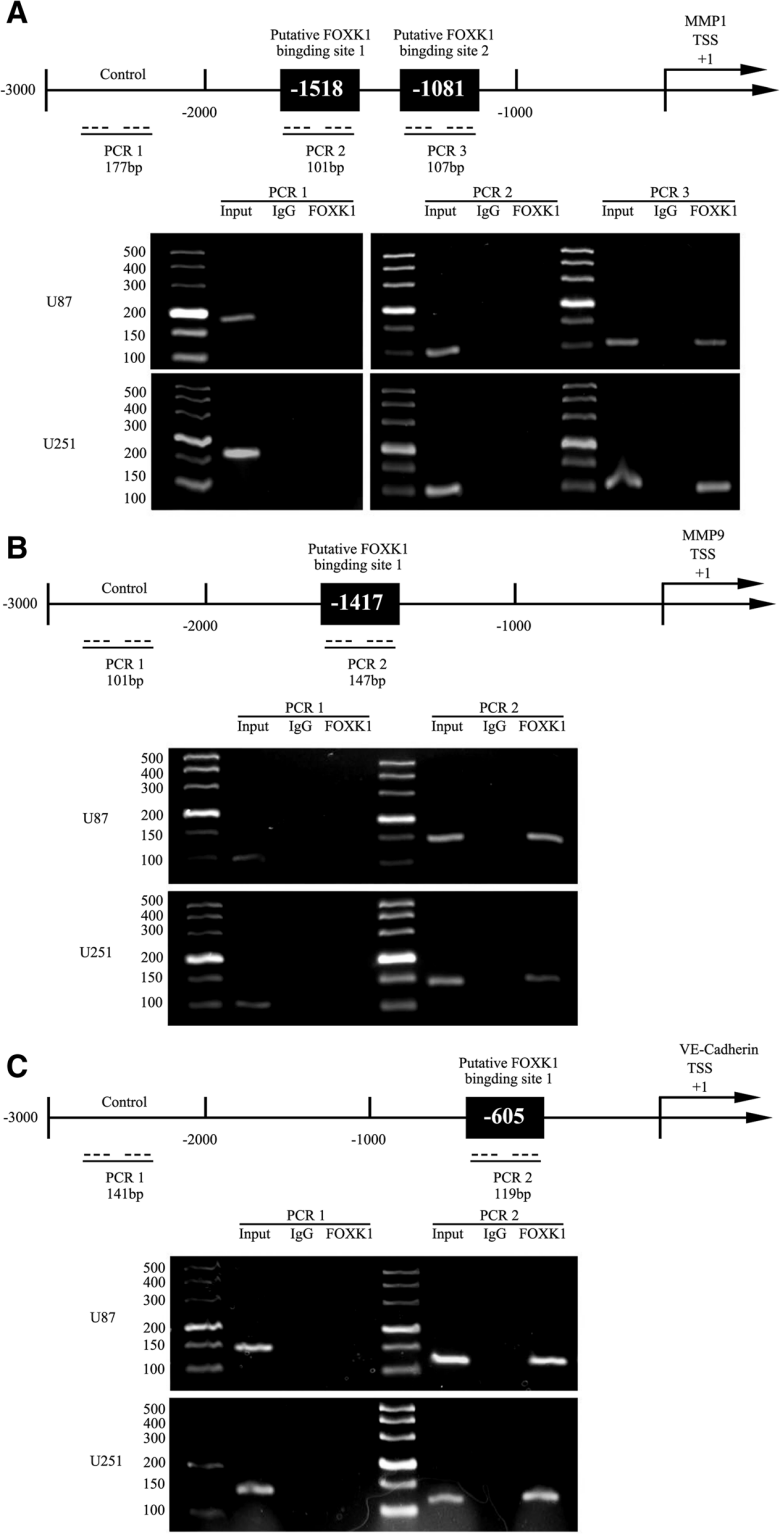
FOXK1 bound to the promoters of MMP1, MMP9 and VE-Cadherin in U87 and U251 cells. **a** Schematic representation of the MMP1 promoter region in 3000 bp upstream of the transcription start site (TSS) designated as + 1. Putative FOXK1 binding sites are shown. Immunoprecipitated DNA was amplified by PCR using rabbit IgG as negative control. **b** Schematic representation of the MMP9 promoter region in 3000 bp upstream of the transcription start site (TSS) designated as + 1. Putative FOXK1 binding sites are shown. Immunoprecipitated DNA was amplified by PCR using rabbit IgG as negative control. **c** Schematic representation of the VE-Cadherin promoter region in 3000 bp upstream of the transcription start site (TSS) designated as + 1. Putative FOXK1 binding sites are shown. Immunoprecipitated DNA was amplified by PCR using rabbit IgG as negative control

By quiring the bioinformatics database (JASPAR), we identified MMP1, MMP9, VE-Cadherin might be probable downstream molecules of FOXK1. Further, we conducted CHIP assays and used MMP1, MMP9, VE-Cadherin promoter sequences according to the DBTSS HOME database (Fig. 6). Two putative FOXK1 binding sites in MMP1, one putative binding site in MMP9 and one binding site in VE-Cadherin were identified. Besides, PCR was used to amplified the 2000 bp upstream regions as negative control. CHIP results revealed that FOXK1 directly bound to the promoters of MMP1, MMP9, VE-Cadherin.

### ZRANB2 knockdown combined with SNHG20 knockdown suppressed tumor growth, generated the longest survival time and yielded the lowest VM formation in vivo

**Fig. 7:**
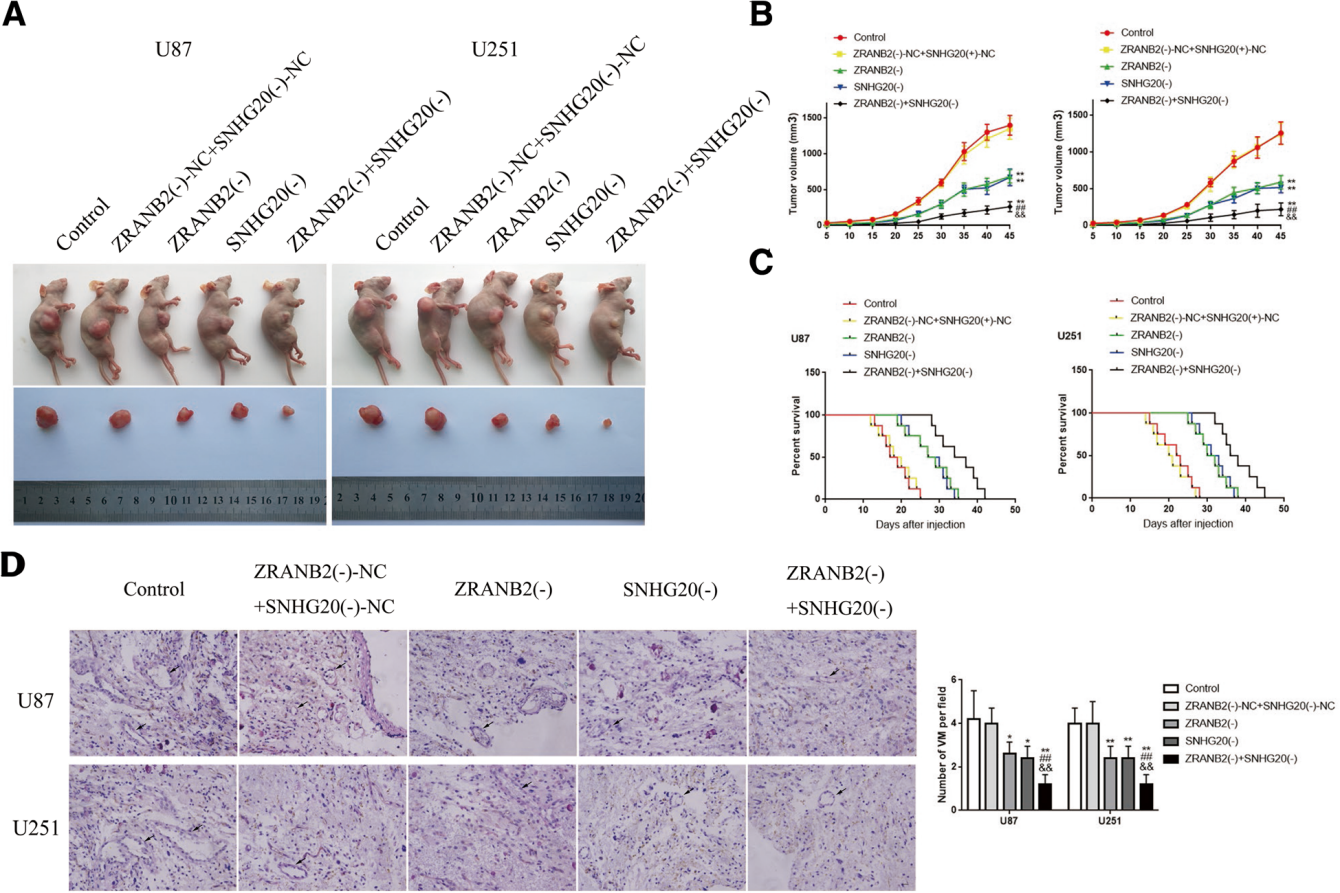
The stable expressing cells were used for tumor xenografts study in vivo. **a** The nude mice sample tumor from respective group was shown. **b** Tumor growth curves in nude mice were shown. Tumor volume was calculated every five days after injection and tumor was excised after 45 days; data are presented as mean ± SD (*n* = 8, each group). ^**^P < 0.01 vs. ZRANB2(−)-NC + SNHG20(−)-NC group, ^##^*P* < 0.01 vs. ZRANB2(−) group, ^&&^*P* < 0.01 vs. SNHG20(−) group. **c** The survival curves of nude mice that were injected into the right striatum were shown *(n* = 8, each group). **d** CD34-PAS staining was used to detect the VM in xenografted tumor; data are presented as mean ± SD (*n* = 5, each group). ^*^P < 0.05 vs. ZRANB2(−)-NC + SNHG20(−)-NC group, ^**^P < 0.01 vs. ZRANB2(−)-NC + SNHG20(−)-NC group, ^##^*P* < 0.01 vs. ZRANB2(−) group, ^&&^*P* < 0.01 vs. SNHG20(−) group. Scale bars indicate 25 μm

In vivo xenograft model experiment was used to verify the above findings. As shown in Fig. 7a-c, ZRANB2(−), SNHG20(−) and ZRANB2(−) + SNHG20(−) group had the smaller tumor size and longer survival time than ZRANB2(−)-NC + SNHG20(−) group. In addition, ZRANB2(−) + SNHG20(−) group gained the smallest tumor size and the longest survival time among all the groups. Last, we conducted the CD34-PAS dual-staining to reveal the VM in every group. As shown in Fig. 7d, ZRANB2(−), SNHG20(−) and ZRANB2(−) + SNHG20(−) group had lower VM density than ZRANB2(−)-NC + SNHG20(−) group. Besides, ZRANB2(−) + SNHG20(−) group had the lowest VM density among all the groups.

## Discussion

**Fig. 8:**
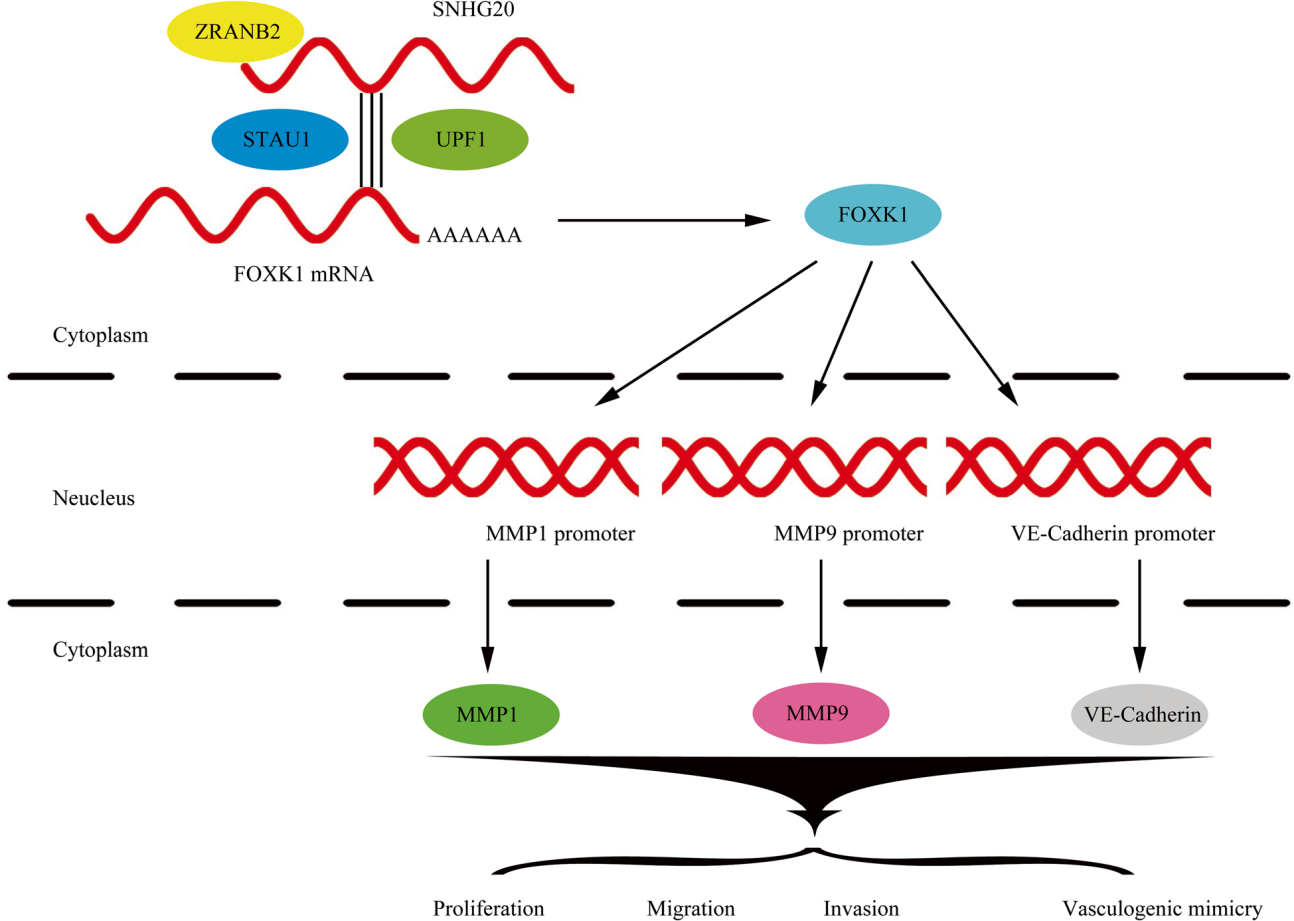
The schematic illusion of interactions between ZRANB2, SNHG20 and FOXK1 in glioma

ZRANB2 is a highly conserved alternative splicing regulatory molecule as an RBP, and its zinc finger structure is the binding region of RNA [25]. This study demonstrates for the first time that RBP-ZRANB2 is highly expressed in glioma tissues and cells. ZRANB2 knockdown inhibits the proliferation, migration, invasion and VM of glioma cells. Inhibition of ZRANB2 reduces the stability and expression of SNHG20. Down-regulated SNHG20 reduces the transcription factor FOXK1 mRNA degradation by targeting its 3’UTR through the SMD pathway, thereby increasing the expression of FOXK1. FOXK1 down-regulates the levels of VM-related molecular MMP1, MMP9, VE-Cadherin by binding to their promoters and inhibited the VM formation in glioma cells. The ZRANB2/SNHG20/FOXK1 axis is presented in Fig. 8 schematically.

RBP exerts a variety of biological regulatory functions in tumors, and binding to lncRNA or mRNA and enhancing its stability had been reported in many studies [26]. This study found that ZRANB2 specifically bound to SNHG20. More importantly, ZRANB2 did not affect the nascent SNHG20 but increased the stability of SNHG20 by prolonging its half-life. Consequently, ZRANB2 promoted VM formation in glioma cells at least by increasing the stability of SNHG20. The similar in vitro and in vivo approaches can be found in previous research [27]. Similar to the results of our study, RBP serine and arginine rich splicing factor 1 (SRSF1) increases the stability of the DNA repair gene DNA ligase 1 (LIG1) mRNA in non-small cell lung cancer and enhances its expression in a mTOR-dependent manner, thereby promoting the proliferation of non-small cell lung cancer cells and attenuated the apoptosis [28]. Poly(rC) binding protein 1 (PCBP1) protein binds to p27 mRNA and enhanced its stability in ovarian cancer cells, thereby enhancing p27 mRNA translation, inhibiting cell cycle and promoting apoptosis [29]. ELAV like RNA binding protein 1 (ELAVL1, HuR) is highly expressed in osteosarcoma, and by binding to high mobility group AT-hook 1 (HMGA1) 3’UTR increases the stability and expression of HMGA1 mRNA, promoting the progression of osteosarcoma [30]; HuR also binds and increases the expression of lncRNA SRY (sex determining region Y)-box 5 (Sox5) and promotes the migration and invasion of human tongue squamous cell carcinoma [31]. Lin-28 homolog B (LIN28B) protein binds and allows translation of AKT serine/threonine kinase 2 (AKT2) mRNA in ovarian cancer to inhibit apoptosis of ovarian cancer cells via AKT2/FOXO3A/BIM axis [32]; LIN28B also promotes proliferation and migration of high-grade serous ovarian cancer by binding to NEAT1 and enhancing its stability [33].

Early studies have shown that lncRNA regulates the progression of a variety of tumors including epigenetic regulation, transcriptional regulation, post-transcriptional regulation, translational regulation [34–36]. For example, HOX transcript antisense RNA (HOTAIR) inhibits cyclin dependent kinase inhibitor 2A (CDKN2A) promoter activity by DNA methylation in osteosarcoma cells [37]. Moreover, metastasis associated lung adenocarcinoma transcript 1 (Malat1) regulates SP1 downstream protein expression in lung cancer by enhancing the stability and transcriptional activity of SP1 [38]. In gastric cancer, knockdown of EGFR-AS1 reduces EGFR expression by decreasing the stability of EGFR mRNA [39]. AFAP1-AS1 regulates the expression of miR-423-5p in nasopharyngeal carcinoma through competitive endogenous RNA pathway [40]. H19 acts as a precursor of miR-673 in breast cancer to enhance breast cancer cell proliferation and invasion [41]. Similarly, our study finds that SNHG20 is highly expressed in glioma tissues and cells, and knockdown of SNHG20 reduces VM of glioma by reducing the expression of VM-related molecular MMP1, MMP9, VE-Cadherin. The results suggest that SNHG20 plays as oncogenic role in gliomas. Earlier studies also prove SNHG20 as tumor-suppressive gene in other tumors consistent with our findings. For example, knockdown of SNHG20 inhibits the expression of β-catenin, inhibits the Wnt/β-catenin signaling pathway, and increased the apoptosis of ovarian cancer cells [42]. SNHG20 is up-regulated in osteosarcoma tissues and cells and knockdown of SNHG20 up-regulates the expression of apoptosis-related protein Bax, decreases the expression of Bcl-2, inhibits the activity of caspase-3 and caspase-9, and promotes the apoptosis of osteosarcoma cells [43]. SNHG20 is also highly expressed in non-small cell lung cancer, down-regulates the expression of the cell cycle-associated protein kinase inhibitor P21, which has been shown to directly bind to and affect G1/S transformation related kinases such as CyclinD/CDK4, CyclinD/CDK6, and CyclinE/ CDK2, and promotes tumor cell proliferation, induces apoptosis, and leads to cell cycle arrest [14]. In addition, in colorectal cancer, high expression of SNHG20 is an important independent risk factor for colorectal cancer patients and silencing SNHG20 increases p21 mRNA and protein expression levels and reduced CyclinA1 expression [17]. SNHG20 is highly expressed in cervical cancer and promotes proliferation and invasion of cervical cancer cells through the miR-140-5p/ADAM10/MEK-ERK axis [16]. In addition, studies find that SNHG20 regulates breast cancer cell proliferation, migration and invasion in breast cancer through the miR-495 / HER2 axis [15]. In bladder cancer, SNHG20 promotes bladder cancer cell proliferation and inhibits apoptosis through the Wnt/β-catenin pathway [44]. Moreover, SNHG20 is up-regulated in hepatocellular carcinoma and can be used as an independent prognostic factor [45].

Staufen1 (STAU1)-mediated mRNA decay is one way of mRNA degradation. For instance, lncRNA-TINCR can form TINCR-STAU1 complex to degrade cyclin dependent kinase inhibitor 2B (CDKN2B) mRNA through the SMD pathway in gastric cancer [46]. During somatic differentiation, the differentiation-related protein KRT80 mRNA binds to TINCR and STAU1 via its Alu elements and finally is degraded by UPF1 [47]. Besides, the paired box 3 (PAX3) mRNA whose product is a protein essential for myoblast differentiation is also degraded by the SMD pathway [48]. In addition, the SMD pathway mediates the degradation of growth associated protein 43 (GAP43) mRNA in mouse C2C12 myoblasts [49]. In our study, dual luciferase assay and RNA-IP assay demonstrate that SNHG20 and FOXK1 mRNA bind to STAU1 through the SBS site. Knockdown of STAU1 or UPF1 prolongs the half-life of FOXK1 mRNA and enhanced its stability expression. This study demonstrates for the first time that SNHG20 promotes the degradation of FOXK1 mRNA through the SMD pathway and regulates the VM formation in gliomas.

FOXK1 belongs to transcription factor of forkhead family and plays various biological functions in different tumors. For example, in colorectal cancer, FOXK1 acts as a DVL-interacting protein, and transfers DVL-related proteins into the nucleus with FOXK2, which positively regulates the Wnt/β-catenin signaling pathway [20]. In lung cancer, FOXK1 acts as a downstream target of circMAN2B2/miR-1275 and plays a role as an oncogene [50]. It is reported that overexpression of FOXK1 inhibits apoptosis of esophageal cancer cells and promoted cell proliferation and migration [51]. However, the expression level of FOXK1 in breast cancer is significantly lower than that in adjacent lung cancer tissues; FOXK1 knockdown decreases the expression of E-cadherin and increases the expression of N-cadherin, which suggests that FOXK1 inhibits the invasion of breast cancer cells by inhibiting epithelial-mesenchymal transition (EMT) [21]. Our study finds that FOXK1 is down-regulated in glioma tissues and cells and is negatively correlated with pathological grade of glioma. Overexpression of FOXK1 significantly inhibits the proliferation, migration and invasion of glioma cells. FOXK1 significantly reduces VM-related molecules MMP1, MMP9 and VE-Cadherin and reduced VM in glioma cells. In consideration of the different reports of FOXK1 as an oncogene or tumor suppressor in different tumors, we refer to the TCGA database for FOXK1 expression in glioma, as well as survival curves (Fig. 4c-d). Compared with tissues, FOXK1 expresses lower in glioma tissues, but the number of cases in NBTs is only 5 and the results shows no significant difference. However, survival curve analysis shows that glioma cases of FOXK1 high-expression have longer survival than that with FOXK1 low-expression (*P* < 0.0001). Statistical analysis of the TCGA database partly caters to the results of this study.

Moreover, further researches are needed to check for the results on tumor cells issued from fresh tumors and maintained in culture. Moreover, whether knockdown and overexpression of these molecules in normal cells could induce similar VM-related effects needs further studies.

## Conclusions

In summary, our study found for the first time that ZRANB2 and SNHG20 are up-regulated in glioma tissues and glioma cells. ZRANB2 bound to SNHG20, and SNHG20 acted on FOXK1 via SMD pathway. Knockdown ZRANB2 reduced the stability of SNHG20, and reduced the degradation of FOXK1 mRNA by SMD pathway subsequently. FOXK1 inhibited transcription by binding to the promoters of MMP1, MMP9 and VE-Cadherin and inhibited the proliferation and migration, invasion and VM formation of glioma cells. This study demonstrated that the ZRANB2/SNHG20/FOXK1 axis played an important role in regulating the expression of VM-related molecules MMP1, MMP9 and VE-Cadherin, and regulated the VM formation of glioma. Our results reveal a new mechanism that identifies novel possible targets which might be useful for future therapeutic interventions.

## Additional file

Additional file 1: **Figure S1.** (A) Typical CD34-PAS dual staining of VM and correlation between ZRANB2 and VM. (B) Expression of ZRANB2 mRNA in NHA, U87, U373, U251 and A172 cells. Data are presented as mean ± SD (*n* = 3, each group). ^**^P < 0.01 vs. NHA group. (C) The efficiency of silencing of ZRANB2 in U373 cells. (D) Three-dimensional culture. Data are presented as mean ± SD (n = 3, each group). ^*^P < 0.05 vs. ZRANB2(−)-NC group. Scale bars indicate 50 μm. (E) The efficiency of silencing of ZRANB2 in A172 cells. (F) Three-dimensional culture. Data are presented as mean ± SD (*n* = 3, each group). ^*^P < 0.05 vs. ZRANB2(−)-NC group. Scale bars indicate 50 μm. (G) The efficiencies of silencing and overexpression of ZRANB2 in U87 and U251 cells. (H) The efficiencies of silencing and overexpression of SNHG20 in U87 and U251 cells. (I) The efficiencies of co-transfection of ZRANB2 and SNHG20 in U87 and U251 cells. (J) The efficiencies of silencing and overexpression of FOXK1 in U87 and U251 cells. (K) The efficiencies of co-transfection of SNHG20 and FOXK1 in U87 and U251 cells. (L) Laminin-5gamma2’ staining in xenografted tumor. Scale bars indicate 25 μm. (M) Ki67 staining in xenografted tumor, data are presented as mean ± SD (*n* = 3, each group). ^*^P < 0.05 vs. ZRANB2(−)-NC + SNHG20(−)-NC group, ^**^P < 0.01 vs. ZRANB2(−)-NC + SNHG20(−)-NC group, ^#^*P* < 0.05 vs. ZRANB2(−) group, ^&^*P* < 0.05 vs. SNHG20(−) group. Scale bars indicate 25 μm. (PDF 3339 kb)

## Abbreviations

ActD: Actinomycin D
CCK-8: Cell Counting Kit-8
CHIP: Chromatin immunoprecipitation
FOXK1: Forkhead box K1
LncRNAs: Long non-coding RNAs
MMP: Matrix metalloproteinase
NBTs: Normal brain tissues
NHA: Normal human astrocyte
PAS: Periodic acid-schiff
qRT-PCR: Quantitative real-time PCR
RNPs: RNA-binding proteins
SBS: STAU1 binding site
SMD: STAU1-mediated mRNA decay
VE-Cadherin: Vascular endothelial cadherin
VM: vasculogenic mimicry
ZRANB2: Zinc-finger RAN-binding domain containing protein 2

## References

[1] Lapointe S, Perry A, Butowski NA (2018). Primary brain tumours in adults. Lancet.

[2] Zhou Q, Liu J, Quan J, Liu W, Tan H, Li W (2018). lncRNAs as potential molecular biomarkers for the clinicopathology and prognosis of glioma: a systematic review and meta-analysis. Gene.

[3] Hombach-Klonisch S, Mehrpour M, Shojaei S, Harlos C, Pitz M, Hamai A (2018). Glioblastoma and chemoresistance to alkylating agents: involvement of apoptosis, autophagy, and unfolded protein response. Pharmacol Ther.

[4] Wick W, Osswald M, Wick A, Winkler F (2018). Treatment of glioblastoma in adults. Ther Adv Neurol Disord.

[5] Chen YS, Chen ZP (2014). Vasculogenic mimicry: a novel target for glioma therapy. Chin J Cancer.

[6] Kong Z, Yan C, Zhu R, Wang J, Wang Y, Wang Y (2018). Imaging biomarkers guided anti-angiogenic therapy for malignant gliomas. Neuroimage Clin.

[7] Skelton WT, Castagno J, Cardenas-Goicoechea J, Daily K, Yeung A, Markham MJ (2018). Bevacizumab eligibility in patients with metastatic and recurrent cervical Cancer: a retrospective review. Clin Med Insights Oncol.

[8] Maniotis AJ, Folberg R, Hess A, Seftor EA, Gardner LM, Pe'er J (1999). Vascular channel formation by human melanoma cells in vivo and in vitro: vasculogenic mimicry. Am J Pathol.

[9] Hong S (2017). RNA binding protein as an emerging therapeutic target for Cancer prevention and treatment. J Cancer Prev.

[10] Yang YH, Markus MA, Mangs AH, Raitskin O, Sperling R, Morris BJ (2013). ZRANB2 localizes to supraspliceosomes and influences the alternative splicing of multiple genes in the transcriptome. Mol Biol Rep.

[11] Ohte S, Kokabu S, Iemura S, Sasanuma H, Yoneyama K, Shin M (2012). Identification and functional analysis of Zranb2 as a novel Smad-binding protein that suppresses BMP signaling. J Cell Biochem.

[12] Sun W, Yang Y, Xu C, Guo J (2017). Regulatory mechanisms of long noncoding RNAs on gene expression in cancers. Cancer Genet.

[13] Liu J, Lu C, Xiao M, Jiang F, Qu L, Ni R (2017). Long non-coding RNA SNHG20 predicts a poor prognosis for HCC and promotes cell invasion by regulating the epithelial-to-mesenchymal transition. Biomed Pharmacother.

[14] Chen Z, Chen X, Chen P, Yu S, Nie F, Lu B (2017). Long non-coding RNA SNHG20 promotes non-small cell lung cancer cell proliferation and migration by epigenetically silencing of P21 expression. Cell Death Dis.

[15] Guan YX, Zhang MZ, Chen XZ, Zhang Q, Liu SZ, Zhang YL. Lnc RNA SNHG20 participated in proliferation, invasion, and migration of breast cancer cells via miR-495. J Cell Biochem. 2018;119:7971-81. 10.1002/jcb.26588 29236315

[16] Guo H, Yang S, Li S, Yan M, Li L, Zhang H (2018). LncRNA SNHG20 promotes cell proliferation and invasion via miR-140-5p-ADAM10 axis in cervical cancer. Biomed Pharmacother.

[17] Li C, Zhou L, He J, Fang XQ, Zhu SW, Xiong MM (2016). Increased long noncoding RNA SNHG20 predicts poor prognosis in colorectal cancer. BMC Cancer.

[18] Imamachi N, Tani H, Akimitsu N (2012). Up-frameshift protein 1 (UPF1): multitalented entertainer in RNA decay. Drug Discov Ther.

[19] Park E, Maquat LE (2013). Staufen-mediated mRNA decay. Wiley Interdiscip Rev RNA.

[20] Wang W, Li X, Lee M, Jun S, Aziz KE, Feng L (2015). FOXKs promote Wnt/beta-catenin signaling by translocating DVL into the nucleus. Dev Cell.

[21] Sun T, Wang H, Li Q, Qian Z, Shen C (2016). Forkhead box protein k1 recruits TET1 to act as a tumor suppressor and is associated with MRI detection. Jpn J Clin Oncol.

[22] Lin H, Pan JC, Zhang FM, Huang B, Chen X, Zhuang JT (2015). Matrix metalloproteinase-9 is required for vasculogenic mimicry by clear cell renal carcinoma cells. Urol Oncol.

[23] Sood AK, Seftor EA, Fletcher MS, Gardner LM, Heidger PM, Buller RE (2001). Molecular determinants of ovarian cancer plasticity. Am J Pathol.

[24] Mao XG, Xue XY, Wang L, Zhang X, Yan M, Tu YY (2013). CDH5 is specifically activated in glioblastoma stemlike cells and contributes to vasculogenic mimicry induced by hypoxia. Neuro-Oncology.

[25] Loughlin FE, Mansfield RE, Vaz PM, McGrath AP, Setiyaputra S, Gamsjaeger R (2009). The zinc fingers of the SR-like protein ZRANB2 are single-stranded RNA-binding domains that recognize 5′ splice site-like sequences. Proc Natl Acad Sci U S A.

[26] Wu XS, Wang F, Li HF, Hu YP, Jiang L, Zhang F (2017). LncRNA-PAGBC acts as a microRNA sponge and promotes gallbladder tumorigenesis. EMBO Rep.

[27] Guo J, Cai H, Liu X, Zheng J, Liu Y, Gong W (2018). Long non-coding RNA LINC00339 stimulates glioma Vasculogenic mimicry formation by regulating the miR-539-5p/TWIST1/MMPs Axis. Mol Ther Nucleic Acids.

[28] Martinez-Terroba E, Ezponda T, Bertolo C, Sainz C, Remirez A, Agorreta J, et al. The oncogenic RNA-binding protein SRSF1 regulates LIG1 in non-small cell lung cancer. Lab Investig. 2018. 10.1038/s41374-018-0128-2 30181552

[29] Shi H, Li H, Yuan R, Guan W, Zhang X, Zhang S (2018). PCBP1 depletion promotes tumorigenesis through attenuation of p27(Kip1) mRNA stability and translation. J Exp Clin Cancer Res.

[30] Pan W, Pang J, Ji B, Wang Z, Liu C, Cheng Y (2018). RNA binding protein HuR promotes osteosarcoma cell progression via suppressing the miR-142-3p/HMGA1 axis. Oncol Lett.

[31] Wang L, Ye S, Wang J, Gu Z, Zhang Y, Zhang C (2017). HuR stabilizes lnc-Sox5 mRNA to promote tongue carcinogenesis. Biochemistry (Mosc).

[32] Lin X, Shen J, Dan P, He X, Xu C, Chen X (2018). RNA-binding protein LIN28B inhibits apoptosis through regulation of the AKT2/FOXO3A/BIM axis in ovarian cancer cells. Signal Transduct Target Ther.

[33] Yong W, Yu D, Jun Z, Yachen D, Weiwei W, Midie X (2018). Long noncoding RNA NEAT1, regulated by LIN28B, promotes cell proliferation and migration through sponging miR-506 in high-grade serous ovarian cancer. Cell Death Dis.

[34] Dang Y, Wei X, Xue L, Wen F, Gu J, Zheng H (2018). Long non-coding RNA in glioma: target miRNA and signaling pathways. Clin Lab.

[35] Diamantopoulos MA, Tsiakanikas P, Scorilas A (2018). Non-coding RNAs: the riddle of the transcriptome and their perspectives in cancer. Ann Transl Med.

[36] Xu T, Lin CM, Cheng SQ, Min J, Li L, Meng XM (2018). Pathological bases and clinical impact of long noncoding RNAs in prostate cancer: a new budding star. Mol Cancer.

[37] Li X, Lu H, Fan G, He M, Sun Y, Xu K (2017). A novel interplay between HOTAIR and DNA methylation in osteosarcoma cells indicates a new therapeutic strategy. J Cancer Res Clin Oncol.

[38] Li S, Ma F, Jiang K, Shan H, Shi M, Chen B (2018). Long non-coding RNA metastasis-associated lung adenocarcinoma transcript 1 promotes lung adenocarcinoma by directly interacting with specificity protein 1. Cancer Sci.

[39] Hu J, Qian Y, Peng L, Ma L, Qiu T, Liu Y (2018). Long noncoding RNA EGFR-AS1 promotes cell proliferation by increasing EGFR mRNA stability in gastric Cancer. Cell Physiol Biochem.

[40] Lian Y, Xiong F, Yang L, Bo H, Gong Z, Wang Y (2018). Long noncoding RNA AFAP1-AS1 acts AS a competing endogenous RNA of miR-423-5p to facilitate nasopharyngeal carcinoma metastasis through regulating the rho/Rac pathway. J Exp Clin Cancer Res.

[41] Vennin C, Spruyt N, Dahmani F, Julien S, Bertucci F, Finetti P (2015). H19 non coding RNA-derived miR-675 enhances tumorigenesis and metastasis of breast cancer cells by downregulating c-Cbl and Cbl-b. Oncotarget.

[42] He S, Zhao Y, Wang X, Deng Y, Wan Z, Yao S, et al. Up-regulation of long non-coding RNA SNHG20 promotes ovarian cancer progression via Wnt/beta-catenin signaling. Biosci Rep. 2018. 10.1042/BSR20170681. 10.1042/BSR20170681 PMC5754315 29101241

[43] Wang W, Luo P, Guo W, Shi Y, Xu D, Zheng H (2018). LncRNA SNHG20 knockdown suppresses the osteosarcoma tumorigenesis through the mitochondrial apoptosis pathway by miR-139/RUNX2 axis. Biochem Biophys Res Commun.

[44] Zhao Q, Gao S, Du Q, Liu Y (2018). Long non-coding RNA SNHG20 promotes bladder cancer via activating the Wnt/beta-catenin signalling pathway. Int J Mol Med.

[45] Zhang D, Cao C, Liu L, Wu D (2016). Up-regulation of LncRNA SNHG20 predicts poor prognosis in hepatocellular carcinoma. J Cancer.

[46] Xu TP, Wang YF, Xiong WL, Ma P, Wang WY, Chen WM (2017). E2F1 induces TINCR transcriptional activity and accelerates gastric cancer progression via activation of TINCR/STAU1/CDKN2B signaling axis. Cell Death Dis.

[47] Kretz M, Siprashvili Z, Chu C, Webster DE, Zehnder A, Qu K (2013). Control of somatic tissue differentiation by the long non-coding RNA TINCR. Nature.

[48] Gong C, Kim YK, Woeller CF, Tang Y, Maquat LE (2009). SMD and NMD are competitive pathways that contribute to myogenesis: effects on PAX3 and myogenin mRNAs. Genes Dev.

[49] Kim YK, Furic L, Parisien M, Major F, DesGroseillers L, Maquat LE (2007). Staufen1 regulates diverse classes of mammalian transcripts. EMBO J.

[50] Ma X, Yang X, Bao W, Li S, Liang S, Sun Y (2018). Circular RNA circMAN2B2 facilitates lung cancer cell proliferation and invasion via miR-1275/FOXK1 axis. Biochem Biophys Res Commun.

[51] Chen D, Wang K, Li X, Jiang M, Ni L, Xu B (2017). FOXK1 plays an oncogenic role in the development of esophageal cancer. Biochem Biophys Res Commun.

