# Supplementary figures and images for: ZRANB2/SNHG20/FOXK1 Axis regulates Vasculogenic mimicry formation in glioma
Source: J Exp Clin Cancer Res. 2019 Feb 11;38:68. doi: 10.1186/s13046-019-1073-7 (PMC6371528; doi:10.1186/s13046-019-1073-7)

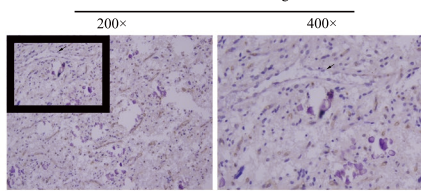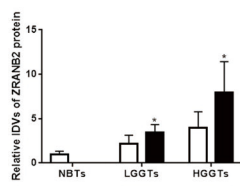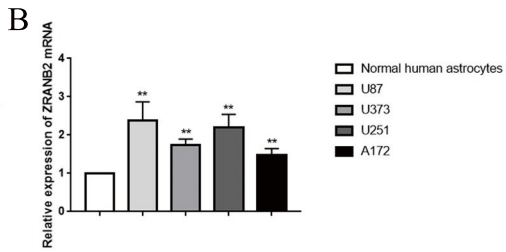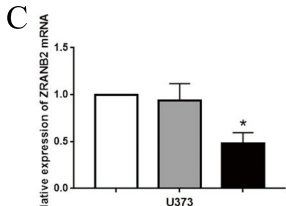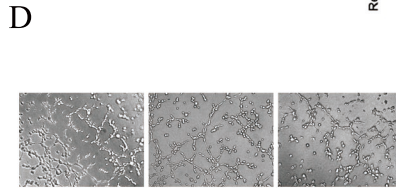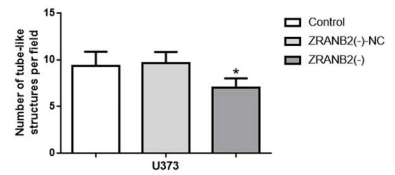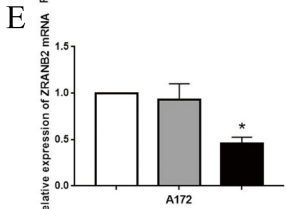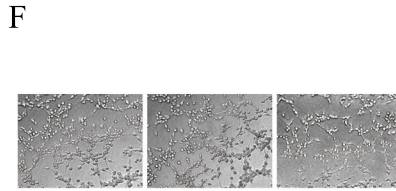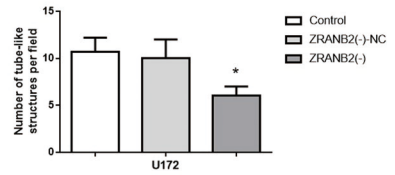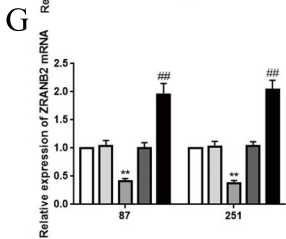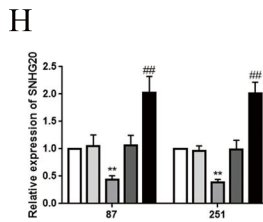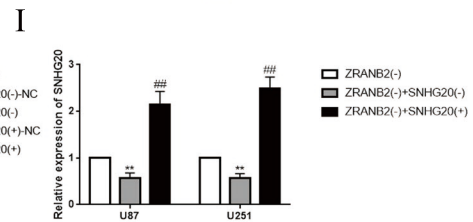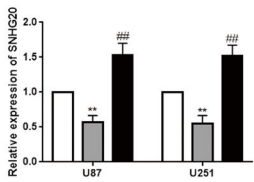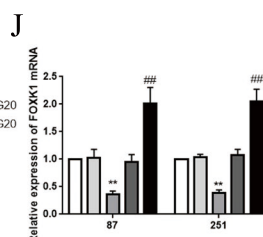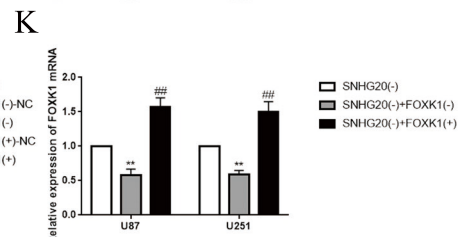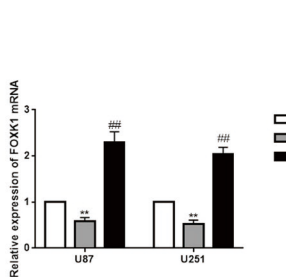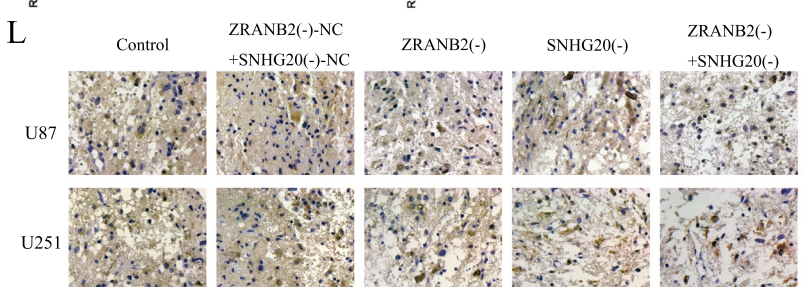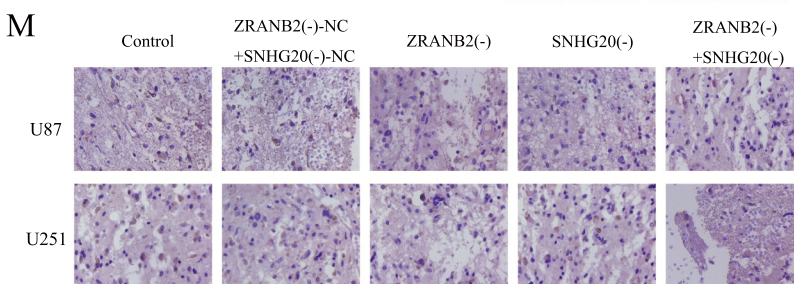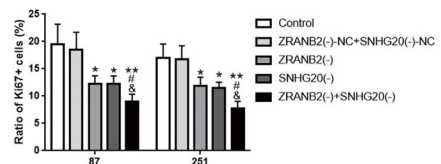

Supplement: Supplementary file 1 — Figure S1. (A) Typical CD34-PAS dual staining of VM and correlation between ZRANB2 and VM. (B) Expression of ZRANB2 mRNA in NHA, U87, U373, U251 and A172 cells. Data are presented as mean ± SD (n = 3, each group). **P < 0.01 vs. NHA group. (C) The efficiency of silencing of ZRANB2 in U373 cells. (D) Three-dimensional culture. Data are presented as mean ± SD (n = 3, each group). *P < 0.05 vs. ZRANB2(−)-NC group. Scale bars indicate 50 μm. (E) The efficiency of silencing of ZRANB2 in A172 cells. (F) Three-dimensional culture. Data are presented as mean ± SD (n = 3, each group). *P < 0.05 vs. ZRANB2(−)-NC group. Scale bars indicate 50 μm. (G) The efficiencies of silencing and overexpression of ZRANB2 in U87 and U251 cells. (H) The efficiencies of silencing and overexpression of SNHG20 in U87 and U251 cells. (I) The efficiencies of co-transfection of ZRANB2 and SNHG20 in U87 and U251 cells. (J) The efficiencies of silencing and overexpression of FOXK1 in U87 and U251 cells. (K) The efficiencies of co-transfection of SNHG20 and FOXK1 in U87 and U251 cells. (L) Laminin-5gamma2’ staining in xenografted tumor. Scale bars indicate 25 μm. (M) Ki67 staining in xenografted tumor, data are presented as mean ± SD (n = 3, each group). *P < 0.05 vs. ZRANB2(−)-NC + SNHG20(−)-NC group, **P < 0.01 vs. ZRANB2(−)-NC + SNHG20(−)-NC group, #P < 0.05 vs. ZRANB2(−) group, &P < 0.05 vs. SNHG20(−) group. Scale bars indicate 25 μm. (PDF 3339 kb) [file 13046_2019_1073_MOESM1_ESM.pdf]
